# Multi-Epoch Differential Pseudorange Joint Positioning Using GNSS Signals and Terrestrial Cellular Signals-of-Opportunity

**DOI:** 10.3390/s25185800

**Published:** 2025-09-17

**Authors:** Pei Zhang, Tian Jin, James Chakwizira, Yuchen Wang

**Affiliations:** 1 School of Electronic and Information Engineering, Beihang University, Beijing 100191, China; by2002149@buaa.edu.cn; 2 The Faculty of Science, Engineering and Agriculture, University of Venda, Thohoyandou 0950, South Africa; james.chakwizira@univen.ac.za; 3 The South Africa-China Transport Co-Operation Centre, Pretoria 0044, South Africa

**Keywords:** global navigation satellite systems, cellular signals of opportunity, fusion positioning, pseudorange single difference at multi-epoch, low observability

## Abstract

When the global navigation satellite systems (GNSSs) are unavailable, cellular signals of opportunity (SOPs) can be used to achieve positioning. However, in low observability environments where both GNSS signals and cellular SOPs are less than 2, the current research on cellular SOPs–GNSS signals fusion positioning faces challenges regarding the difficulty in precise position initialization and spatiotemporal uncertainty. To address these issues, a cellular SOPs and GNSS signals fusion positioning model by the pseudorange single difference at multi-epoch (PSDM) is proposed. The spatiotemporal uncertainty of fusion positioning is solved by differential pseudorange. Then, to solve the problem of difficult precise location initialization during the differential pseudorange positioning process, a pseudo-linearization closed-form method was derived, and its limitations were analyzed. Moreover, the pseudo-linearization equation was reconstructed. Based on this, a constrained multi-step weighted least squares (CMWLS) method is proposed that reduces the impact of noise on the PSDM positioning models and improves global convergence. According to the simulation and field test results, the proposed fusion positioning method shows good positioning performance in low-observability environments. For urban positioning in such environments, this study provides a new solution strategy and avoids the requirement of the prior information of the receiver’s initial position for positioning.

## 1. Introduction

Global navigation satellite systems (GNSSs) have been used in various fields [1,2]. When GNSS signals are obstructed in urban areas, they are insufficient, making the positioning is unusable [3]. Signals of opportunity (SOPs) refer to non-navigation signals, such as cellular base station (BS) signals and low earth orbit satellite signals [4,5,6,7,8,9]. In GNSS-challenged environments, SOPs can be used as a backup positioning system. Cellular SOP is becoming increasingly popular in urban SOP positioning [10,11,12,13,14,15,16,17,18,19,20,21,22].

In recent years, research on fusion positioning using cellular SOPs and GNSS signals based on pseudorange measurement has been popular [23,24]. There are many studies on pseudorange multipath mitigation that have achieved good performance [25,26]. However, current pseudorange positioning methods require sufficient signal sources.

Considering the asynchrony of BS clocks and the obstruction of BS signals, a low observability environment is defined as a situation where the number of both line-of-sight GNSS satellites and BS signals does not exceed 2. In such environments, the current pseudorange-based fusion studies have poor positioning accuracy and do not have an effective solution strategy. Therefore, it is important to conduct research on cellular SOP–GNSS pseudorange fusion positioning in urban low-observability environments.

The main challenge of cellular SOP–GNSS fusion under low observability is that it is difficult to obtain accurate prior information of the receiver initial position due to insufficient visible GNSS signals, which leads to the problem of difficult position initialization and easy divergence in traditional cellular SOP–GNSS fusion positioning methods. Below, we will analyze the reason for this from two perspectives. Firstly, from the signal perspective, due to the wide coverage range of GNSS signals, the impact of the initial guess on GNSS positioning can be almost negligible. However, the coverage area of cellular BS signals is relatively small in urban environments, and the initial pseudorange estimation error at the hundred meter or kilometer level accounts for a larger proportion of the actual pseudorange measurements. Therefore, cellular SOPs positioning requires accurate initial position information; otherwise, the positioning may fail to converge and may even diverge. From the perspective of positioning models, the clock synchronization conditions between different BSs do not meet the requirements of SOP positioning, and BSs do not broadcast accurate clock synchronization information [27]. Therefore, the different BS clocks are asynchronous. Considering this problem, current research usually uses Kalman filtering (KF) models to achieve cellular SOP–GNSS fusion positioning [28,29,30]. However, the positioning models based on KF also requires precise initialization information to avoid filtering divergence in fusion positioning. Based on the above analysis, the fusion of a few GNSS signals and cellular SOPs for positioning still requires relatively accurate prior information on the initial position of the receiver. In low-observability environments with few GNSS signals, it is difficult to obtain precise position initialization information. Therefore, cellular SOP–GNSS fusion positioning encounters the problem of difficulty in position initialization, and the current research on fusion positioning has not effectively solved this problem.

In addition to Kalman filtering, least squares estimation is also a commonly used fusion positioning method. However, the least squares positioning models based on pseudorange measurements, such as time of arrival (TOA) and time difference of arrival (TDOA), cannot eliminate asynchronous BS clock bias. The positioning equation system of TDOA and TOA may be rank deficient, making it impossible to obtain a unique non-zero solution. Fortunately, although the clock bias of the BS is asynchronous, it has relative stability over a short time period. The clock drift of the BS can be almost ignored in a short period of time [31]. Hence, the pseudorange single difference at multi-epoch (PSDM) can be used to achieve cellular SOP–GNSS fusion positioning based on least squares estimation in this paper. Unlike TDOA, the PSDM refers to the pseudorange difference between different epochs and a specific epoch of the same receiver. Therefore, the PSDM positioning model can eliminate the asynchronous BS clock bias. However, the PSDM measurement model is still nonlinear. When there are insufficient GNSS signals and cellular SOPs, the Taylor linearization method for PSDM positioning still requires a good initial guess; otherwise, the positioning may diverge.

In terms of localization by TDOA, Chan et al. proposed the classic two-step weighted least squares (TWLS) method [32]. TWLS is a closed-form solution method that offers low computational complexity and does not require the receiver’s initial position. Therefore, the closed-form solution method based on pseudo linearization for PSDM positioning is attractive. In recent years, there have been many research achievements that have improved the TWLS method [33,34,35]. However, this method cannot be effectively applied in real low-observability environments due to the demand for more signal sources and high noise sensitivity.

Considering that the receiver is located on the surface of the Earth, K. C. Ho et al. improved the TWLS method by establishing constraints based on the Earth model [36]. However, the Earth is not a sphere, so using the radius of the Earth as a distance is inaccurate. Pei et al. proposed an improved TWLS method via the ellipsoid model and reduced the impact of noise on positioning [37]. This is currently a state-of-the-art study. However, this method requires prior knowledge of the receiver height and numerous number of signals.

To address the above challenges, the main contributions of this work are as follows:

(1) A PSDM positioning model is established to achieve the cellular SOP–GNSS fusion positioning in low observability environments. In this model, the asynchronous BS clock bias is eliminated through the differential pseudorange between different epochs and the initial epoch.
(2) A pseudo-linearization method for PSDM positioning is derived. Different from filtering positioning, this method can obtain a closed-form algebraic solution and avoid the requirement on initial position priors. In addition, the shortcomings of the pseudo-linearization method were analyzed in detail.
(3) In terms of the limitations of the above pseudo linearization method, a constrained multi-step weighted least squares (CMWLS) method for PSDM positioning is proposed and derived. In this method, the pseudo linearization matrix is reconstructed by separating variables, the spatial constraint is established by the mapping relationship between the Earth ellipsoid model and the receiver position, and the BS height can be used as prior information to initialize the constraint conditions. This method reduces the minimum required number of signals, mitigates the problem of noise pollution in pseudo linear processes, and improves the global convergence in positioning.
(4) The convergence of the PSDM model and the positioning performance of the proposed algorithm are analyzed. Then, the influences of the number of visible signals and observation epoch on the proposed positioning method are analyzed. Finally, the proposed method is verified through actual experiments.

Overall, the innovation of this paper is that a PSDM positioning model is proposed to achieve cellular SOP–GNSS fusion in low-observable environments. Aiming to overcome the limitations of the traditional pseudo-linearization method, the pseudo linear matrix is reconstructed, and spatial constraints are established. Then, a CMWLS method for PSDM positioning is proposed to reduce the impact of noise on positioning and improve the convergence. In addition, the proposed positioning method does not rely on the prior receiver’s initial position information.

The remainder of the paper is organized as follows: In Section 2, the measurement models for cellular SOP–GNSS were shown. In Section 3, a PSDM fusion positioning model was established. Then, a pseudo linearization method for PSDM was derived and its shortcomings were analyzed. In Section 4, a CMWLS method for PSDM positioning was derived. In Section 5, the performance of the proposed model and method was analyzed. In Section 6, the experimental results and analysis of different field tests were shown. Finally, the conclusions are given in Section 7.

## 2. Measurement Model

**Figure 1:**
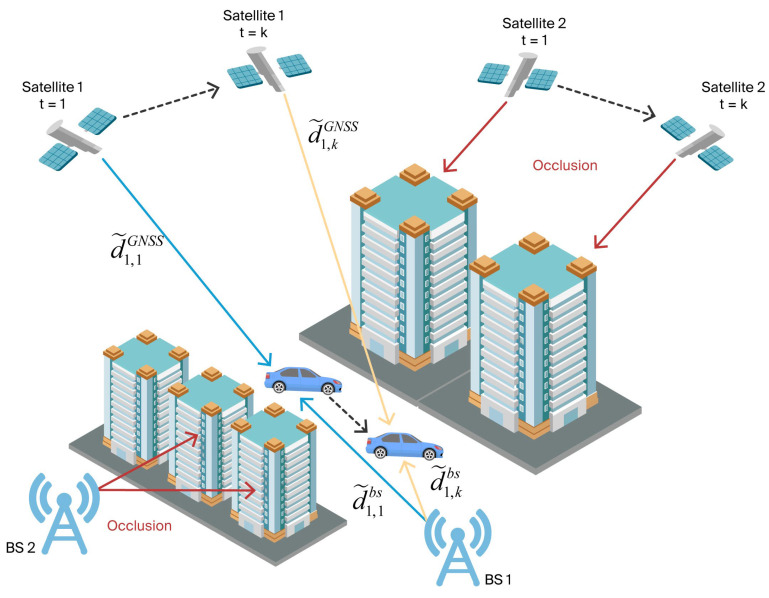
Schematic diagram of cellular SOP–GNSS fusion positioning using PSDM.

For simplicity of notation, the term BS denotes any type of cellular SOP transmitter unless explicitly stated otherwise. The schematic diagram of cellular SOP–GNSS fusion positioning via PSDM is shown in Figure 1. The PSDM measurement models for cellular SOP–GNSS are established in this section. Unlike TOA and TDOA, PSDM measurement in this study refers to the pseudorange difference of the same receiver at different epochs. For convenience, the subscript symbols that appear multiple times in the following text are defined as follows: i is the BS index, j is the satellite index, k is the epoch index, and u is the receiver.

In cellular SOPs positioning, considering the asynchrony of BS clocks, the pseudorange measurement ${\tilde{d}}_{i,k}^{bs}$ is expressed as follows:

(1) $${\tilde{d}}_{i,k}^{bs}=||r_{u,k}-r_{i}^{bs}||+c\delta t_{u}(k)-c\delta t_{bs,i}(k)+\varepsilon_{i,k}^{bs}$$

where c is the speed of light, $r_{u,k}=(x_{u,k},y_{u,k},z_{u,k}{)}^{T}$ is the position of the receiver, k is epoch index, $r_{i}^{bs}=(x_{i}^{bs},y_{i}^{bs},z_{i}^{bs}{)}^{T}$ is the known position of BS, $\left||r_{u,k}-r_{i}^{bs}|\right|$ is the true distance between BS and receiver, $i=1,2,\ldots N^{bs}$, $N^{bs}$ is the number of BSs, ||∗|| is the modulus of a vector, $\delta t_{u}$ is the receiver’s clock bias, $\delta t_{bs,i}$ is the BS’s clock bias, and $\varepsilon_{i,k}^{bs}$ is Gaussian error with variance $\sigma_{bs,i}^{2}(k)$.

Reference [31] states that although the clock bias of each BS is asynchronous and unknown, the clock bias of the same BS has stability in a short period of time (several minutes to ten minutes), that is, the clock drift of the BS is approximately zero. Therefore, the clock bias $\delta t_{bs,i}$ of each BS can be considered constant in a short period of time, such as: $\delta t_{bs,i}=\delta t_{bs,i}(1)\approx \delta t_{bs,i}(k)$. In addition, in most studies related to cellular signal localization, the receiver is set to use a more accurate atomic clock, so the clock bias of the receiver is usually modeled as a first-order polynomial with an initial clock bias $\delta t_{u}(1)$ and clock drift $\dot{\delta }$ [18,30]. Therefore, Equation (1) can be rewritten as follows:

(2) $${\tilde{d}}_{i,k}^{bs}=||r_{u,k}-r_{i}^{bs}||+c(\delta t_{u}(1)+(k-1)T\dot{\delta })-c\delta t_{bs,i}+\varepsilon_{i,k}^{bs}$$

where T is the extraction interval for pseudorange measurement. To eliminate asynchronous BS clock bias, let the first epoch as a reference, the differential pseudorange measurement between the k-th and the 1-th epochs of i-th BS is as:

(3) $$\begin{array}{c} \Delta {\tilde{d}}_{i,k}^{bs}={\tilde{d}}_{i,k}^{bs}-{\tilde{d}}_{i,1}^{bs}=\\ ||r_{u,k}-r_{i}^{bs}||-||r_{u,1}-r_{i}^{bs}||+c(k-1)T\dot{\delta }+\Delta \varepsilon_{i,k}^{bs} \end{array}$$

where $\Delta \varepsilon_{i,k}^{bs}=\varepsilon_{i,k}^{bs}-\varepsilon_{i,1}^{bs}$.

In GNSSs, the positions of the satellites are associated with $\delta t_{u}$. The uncertainty of $\delta t_{u}$ can lead to uncertainty in the satellite position due to the entry of cellular SOPs. Therefore, considering the measurement error of the satellite position, the pseudorange measurement ${\tilde{d}}_{j,k}^{GNSS}$ for GNSS is expressed as:

(4) $$\begin{array}{c} {\tilde{d}}_{j,k}^{GNSS}=||r_{u,k}-({\tilde{r}}_{j,k}^{GNSS}-v_{j,k}^{GNSS}(\delta t_{u}(1)+(k-1)T\dot{\delta }))||+\\ c\delta t_{u}(1)+c(k-1)T\dot{\delta }+\varepsilon_{j,k}^{GNSS} \end{array}$$

where $\left||r_{u,k}-({\tilde{r}}_{j,k}^{GNSS}-\Delta r_{j,k}^{GNSS})|\right|$ is the true range between the receiver and the satellite, $j=1,2,\ldots N^{gnss}$, $N^{gnss}$ is the number of GNSS satellites, ${\tilde{r}}_{j,k}^{GNSS}=r_{j,k}^{GNSS}+\Delta r_{j,k}^{GNSS}$ is the satellite position measurement, $r_{j,k}^{GNSS}$ is the true position, $\Delta r_{j,k}^{GNSS}=v_{j,k}^{GNSS}\delta t_{u}(k)$ is the bias, $v_{j,k}^{GNSS}$ is the velocity of the satellite and it can be obtained from ephemeris, and $\varepsilon_{j,k}^{GNSS}$ is Gaussian noise with variance $\sigma_{gnss,j}^{2}(k)$. Furthermore, the PSDM measurement at k-th epochs of j-th GNSS satellite can be expressed as:

(5) $$\begin{array}{c} \Delta {\tilde{d}}_{j,k}^{GNSS}={\tilde{d}}_{j,k}^{GNSS}-{\tilde{d}}_{j,k}^{GNSS}=\\ \left||r_{u,k}-({\tilde{r}}_{j,k}^{GNSS}-\Delta r_{j,k}^{GNSS})||-||r_{u,1}-({\tilde{r}}_{j,1}^{GNSS}-\Delta r_{j,1}^{GNSS})|\right|\\ +c(k-1)T\dot{\delta }+\Delta \varepsilon_{j,k}^{GNSS} \end{array}$$

where $\Delta \varepsilon_{j,k}^{GNSS}=\varepsilon_{j,k}^{GNSS}-\varepsilon_{j,1}^{GNSS}$.

## 3. PSDM Fusion Positioning Model

In PSDM measurement, asynchronous BS clocks are eliminated. Therefore, in this section, a cellular SOP–GNSS fusion positioning model based on PSDM is established. Then, to avoid the requirement of a model solution on the prior value of the initial position, a closed-form solution method via pseudo-linearization was derived, and its limitations were analyzed.

### 3.1. Establishment of PSDM Fusion Positioning Model

According to Equations (3) and (5), the PSDM measurement equation at the k-th epoch for cellular SOP–GNSS is expressed as:

(6) $$\left[\begin{array}{c} \Delta {\tilde{d}}_{1,k}^{bs}\\ \vdots \\ \Delta {\tilde{d}}_{N^{bs},k}^{bs}\\ \Delta {\tilde{d}}_{1,k}^{GNSS}\\ \vdots \\ \Delta {\tilde{d}}_{N^{gnss},k}^{GNSS} \end{array}\right]=\left[\begin{array}{c} r_{1,k}^{bs}-r_{1,1}^{bs}+c(k-1)T\dot{\delta }+\Delta \varepsilon_{1,k}^{bs}\\ \vdots \\ r_{N^{bs},k}^{bs}-r_{N^{bs},1}^{bs}+c(k-1)T\dot{\delta }+\Delta \varepsilon_{N^{bs},k}^{bs}\\ r_{1,k}^{GNSS}-r_{1,1}^{GNSS}+c(k-1)T\dot{\delta }+\Delta \varepsilon_{1,k}^{GNSS}\\ \vdots \\ r_{N^{\mathrm{gnss}},k}^{GNSS}-r_{N^{gnss},1}^{GNSS}+c(k-1)T\dot{\delta }+\Delta \varepsilon_{N^{gnss},k}^{GNSS} \end{array}\right]$$

where k=2…,L, L is the number of epochs and $r_{i,k}^{bs}=||r_{u,k}-r_{i}^{bs}||$, $r_{j,k}^{GNSS}=||r_{u,k}-({\tilde{r}}_{j,k}^{GNSS}-\Delta r_{j,k}^{GNSS})||$. Convert Equation (6) into the form of a function, the PSDM positioning model composed of the $\left(N^{bs}+N^{gnss})(L-1\right)$ equations are as follows:

(7) $$\tilde{z}=f(x)+\Delta \varepsilon$$

where $\tilde{z}=[({\tilde{z}}_{1,L}^{bs}{)}^{T},\ldots ({\tilde{z}}_{N^{bs},L}^{bs}{)}^{T},({\tilde{z}}_{1,L}^{gnss}{)}^{T}\ldots ({\tilde{z}}_{N^{gnss},L}^{gnss}{)}^{T}{]}_{\left(N^{gnss}+N^{bs})(L-1\right)}^{T}$ is the measurement vector, ${\tilde{z}}_{i,L}^{bs}=[\Delta {\tilde{d}}_{i,2}^{bs}\ldots \Delta {\tilde{d}}_{i,L}^{bs}{]}_{L-1}^{T}$, ${\tilde{z}}_{j,L}^{gnss}=[\Delta {\tilde{d}}_{j,2}^{GNSS}\ldots \Delta {\tilde{d}}_{j,L}^{GNSS}{]}_{L-1}^{T}$, x is the state vector:

(8) $$x=[r_{u,1}^{T},r_{u,2}^{T}\ldots r_{u,L}^{T},c\delta (1),c\dot{\delta }{]}_{3L+2}^{T}$$

The nonlinear function f(x) in Equation (7) is as:

(9) $$f(x)=[f_{1,2}^{bs}(x),\ldots f_{N^{bs},L}^{bs}(x),f_{1,2}^{gnss}(x),\ldots f_{N^{gnss},L}^{gnss}(x){]}^{T}$$

where $f_{i,k}^{bs}(x)=||r_{u,k}-r_{i}^{bs}||-||r_{u,1}-r_{i}^{bs}||+c(k-1)T\dot{\delta }$ and $f_{j,k}^{gnss}(x)=r_{j,k}^{GNSS}-r_{j,1}^{GNSS}+c(k-1)T\dot{\delta }$. The noise vector in (8) is as: $\Delta \varepsilon =[\Delta \varepsilon_{1,2}^{bs},\ldots \Delta \varepsilon_{N^{bs},L}^{bs},\Delta \varepsilon_{1,2}^{GNSS},\ldots \Delta \varepsilon_{N^{gnss},L}^{GNSS}{]}^{T}$.

According to the maximum likelihood estimation theory, solving Equation (7) can be transformed into the following problem:

(10) $$\underset{x}{min}0.5(f(x)-\tilde{z}{)}^{T}Q^{-1}(f(x)-\tilde{z})$$

where $Q=E(\Delta \varepsilon (\Delta \varepsilon {)}^{T})$ and E(∗) is the expectation operator. The Taylor linearization method can be used to solve Equation (10). However, when there are a few visible GNSS signals, if there is no precise receiver initial position, the solution of the Taylor iteration method for solving the PSDM models may fail to converge.

### 3.2. A Pseudo-Linearization Closed-Form Method for PSDM Positioning and Analysis of Its Limitation

Compared to the positioning method based on Taylor linearization, the positioning method based on pseudo-linearization can obtain the state estimation without prior information on the initial position of the receiver. In this subsection, a pseudo-linearization closed-form method for PSDM positioning is derived, and then the limitations of this method are analyzed.

#### 3.2.1. A Pseudo-Linearization Method for PSDM Positioning

Let $d_{i,k}^{bs}=||r_{u,k}-r_{i}^{bs}||$ and $d_{j,k}^{GNSS}=||r_{u,k}-r_{j,k}^{GNSS}||$ be the true range equations without measurement errors, respectively. Substituting $d_{i,k}^{bs}=d_{i,1}^{bs}+\Delta d_{i,k}^{bs}$ and $d_{j,k}^{GNSS}=d_{j,1}^{GNSS}+\Delta d_{j,k}^{GNSS}$ into the true range equation and squaring both sides yields:

(11) $$\begin{array}{c} (\Delta d_{i,k}^{bs}{)}^{2}+2\Delta d_{i,k}^{bs}d_{i,1}^{bs}+(d_{i,1}^{bs}{)}^{2}=\\ r_{u,k}^{T}r_{u,k}-2r_{u,k}^{T}r_{i}^{bs}+(r_{i}^{bs}{)}^{T}r_{i}^{bs} \end{array}$$

(12) $$\begin{array}{c} (\Delta d_{j,k}^{GNSS}{)}^{2}+2\Delta d_{j,k}^{GNSS}d_{j,1}^{GNSS}+(d_{j,1}^{GNSS}{)}^{2}=\\ r_{u,k}^{T}r_{u,k}-2r_{u,k}^{T}r_{j,k}^{GNSS}+(r_{j,k}^{GNSS}{)}^{T}r_{j,k}^{GNSS} \end{array}$$

Let Equation (11) be $N_{k,i}$ and Equation (12) be $Z_{k,j}$. To eliminate the second-order term of the state variable, perform the following operation:

(13) $$N_{k,i}-N_{1,i}-Z_{k,j}+Z_{1,j}$$

Substituting Equations (11) and (12) into Equation (13) yields:

(14) $$\begin{array}{c} \frac{1}{2}((\Delta d_{i,k}^{bs}{)}^{2}-(\Delta d_{j,k}^{GNSS}{)}^{2}+(r_{j,k}^{GNSS}{)}^{T}r_{j,k}^{GNSS}-(r_{j,1}^{GNSS}{)}^{T}r_{j,1}^{GNSS})=\\ -\Delta d_{i,k}^{bs}d_{i,1}^{bs}+\Delta d_{j,k}^{GNSS}d_{j,1}^{GNSS}-r_{u,k}^{T}r_{i}^{bs}+r_{u,1}^{T}r_{i}^{bs}+r_{u,k}^{T}r_{j,k}^{GNSS}-r_{u,1}^{T}r_{j,1}^{GNSS} \end{array}$$

where k=2,3…L. Substituting the PSDM measurement containing errors into Equation (14), the pseudo-linear equations can be constructed by adding auxiliary variables. However, the clock bias and drift cause a sharp increase in the number of auxiliary variables. Therefore, it is necessary to simplify as follows:

(1) At the beginning, the receiver is stationary for a short period of time and the TOAs of cellular SOPs are summarized. Through first-order linear fitting, the receiver clock drift estimate $\hat{\dot{\delta }}$ can be obtained to correct PSDM measurements.
(2) After clock drift correction, the uncertainty of clock bias estimation has a relatively small impact on satellite position, and it is temporarily ignored in satellite position modeling.

The corrected PSDM measurements are $\Delta {\dot{\tilde{d}}}_{j,k}^{GNSS}=\Delta {\tilde{d}}_{j,k}^{GNSS}-c(k-1)T\hat{\dot{\delta }}$ and $\Delta {\dot{\tilde{d}}}_{i,k}^{bs}=\Delta {\tilde{d}}_{i,k}^{bs}-c(k-1)T\hat{\dot{\delta }}$. Substitute the corrected PSDM measurements into Equation (14) and ignore $(\Delta \varepsilon {)}^{2}$, the positioning function composed of $N^{bs}N^{gnss}(L-1)$ linearly independent equations is expressed as:

(15) $${\tilde{C}}_{P}{\tilde{\varepsilon }}_{P}={\tilde{h}}_{P}-{\tilde{G}}_{P}x_{P}$$

where $x_{P}=[r_{u,1}^{T},\ldots r_{u,L}^{T},d_{1,1}^{bs},\ldots d_{N^{bs},1}^{bs},d_{1,1}^{GNSS}\ldots d_{N^{gnss},1}^{GNSS}{]}^{T}$, the measurement is: ${\tilde{h}}_{P}=[h_{2}(1,1),\ldots h_{L}(1,N^{gnss}),\ldots h_{L}(N^{bs},N^{gnss}){]}^{T}$, $h_{k}(i,j)=\frac{1}{2}((\Delta {\dot{\tilde{d}}}_{i,k}^{bs}{)}^{2}-(\Delta {\dot{\tilde{d}}}_{j,k}^{GNSS}{)}^{2}+(r_{j,k}^{GNSS}{)}^{T}r_{j,k}^{GNSS}-(r_{j,1}^{GNSS}{)}^{T}r_{j,1}^{GNSS})$, ${\tilde{C}}_{P}$ is noise matrix, $\varepsilon_{P}=[\Delta \varepsilon_{1,2}^{bs},\ldots \Delta \varepsilon_{N^{bs},L}^{bs},\Delta \varepsilon_{1,2}^{GNSS},\ldots \Delta \varepsilon_{N^{gnss},L}^{GNSS}{]}^{T}$, the coefficient matrix is as:

(16) $${\tilde{G}}_{P}=[G_{1,1}^{T},\ldots G_{1,N^{gnss}}^{T},G_{2,1}^{T},\ldots G_{2,N^{gnss}}^{T},\ldots G_{N^{bs},N^{gnss}}^{T}{]}^{T}$$

where $G_{i,j}=[\begin{array}{ccc} G_{i,j}^{1} & G_{i,j}^{bs} & G_{i,j}^{GNSS} \end{array}]$,

(17) $$\begin{array}{c} G_{i,j}^{1}=\left[\begin{array}{cccc} (r_{i}^{bs}-r_{j,1}^{GNSS}{)}^{T} & (r_{j,2}^{GNSS}-r_{i}^{bs}{)}^{T} & & \\ \vdots & & \ddots & \\ (r_{i}^{bs}-r_{j,1}^{GNSS}{)}^{T} & & & (r_{j,L}^{GNSS}-r_{i}^{bs}{)}^{T} \end{array}\right]\\ G_{i,j}^{bs}=\left[\begin{array}{ccc} 0 & -\Delta {\dot{\tilde{d}}}_{i,2}^{bs} & 0\\ \vdots & \vdots & \vdots \\ 0 & -\Delta {\dot{\tilde{d}}}_{i,L}^{bs} & 0 \end{array}\right],\;G_{i,j}^{GNSS}=\left[\begin{array}{ccc} 0 & \Delta {\dot{\tilde{d}}}_{j,2}^{GNSS} & 0\\ \vdots & \vdots & \vdots \\ 0 & \Delta {\dot{\tilde{d}}}_{j,L}^{GNSS} & 0 \end{array}\right] \end{array}$$

Equation (15) can be solved by weighted least squares (WLS) as:

(18) $${\hat{x}}_{P}=({\tilde{G}}_{P}^{T}Q_{P}^{-1}{\tilde{G}}_{P}{)}^{-1}{\tilde{G}}_{P}Q_{P}^{-1}{\tilde{h}}_{P}$$

where ${\hat{x}}_{P}$ is the state estimation, $Q_{P}=E(({\tilde{C}}_{P}{\tilde{\varepsilon }}_{P})({\tilde{C}}_{P}{\tilde{\varepsilon }}_{P}{)}^{T})$.

#### 3.2.2. Limitation Analysis

According to Equation (18), the estimation accuracy of state ${\hat{x}}_{P}$ can be affected by ${\tilde{G}}_{P}$, ${\tilde{Q}}_{P}$ and ${\tilde{h}}_{P}$. For ${\tilde{h}}_{P}$ and ${\tilde{Q}}_{P}$, the impact of noise is direct. For ${\tilde{G}}_{P}$, according to the structure of ${\tilde{G}}_{P}$ in Equation (17), the elements in columns 3L+1 to $3L+N^{bs}+N^{gnss}$ of ${\tilde{G}}_{P}$ are PSDM measurements, such as $G_{i,j}^{bs}$ and $G_{i,j}^{GNSS}$. This means that the pseudo linearization matrix ${\tilde{G}}_{P}$ is also contaminated by noise. Therefore, as the noise increases, the condition number and singularity of ${\tilde{G}}_{P}$ will increase.

On the other hand, due to the introduction of new auxiliary variables in Equation (15), the matrix ${\tilde{G}}_{P}$ requires more signals to maintain the full rank. For 2D positioning, the number of unknown states is $2L+N^{bs}+N^{gnss}$ in Equation (15), and the number of measurements is $N^{bs}N^{gnss}(L-1)$. The condition for Equation (15) to have a unique non-zero solution is as:

(19) $$N^{bs}N^{gnss}(L-1)\geq 2L+N^{bs}+N^{gnss}$$

In a low-observability environment, when $N^{bs}+N^{gnss}=3$, ${\tilde{G}}_{P}$ is rank deficient, and the above pseudo-linearization method cannot be used to solve the PSDM model. For 3D positioning, the number of unknown states is $3L+N^{bs}+N^{gnss}$ in Equation (15), and the number of measurements is also $N^{bs}N^{gnss}(L-1)$. When $N^{bs}+N^{gnss}<4,$ ${\tilde{G}}_{P}$ is rank deficient, the positioning equation is unsolvable. Therefore, for 3D positioning, at least four visible signals are required.

Overall, the above pseudo-linearization closed-form solution method for PSDM positioning has the following shortcomings:

(1) In ${\tilde{G}}_{P}$, column elements corresponding to auxiliary variables are contaminated by noise. The singularity of ${\tilde{G}}_{P}$ increases and the performance deteriorates significantly in high noise.
(2) Since the addition of auxiliary variables, Equation (15) requires more signals to maintain the full rank of matrix ${\tilde{G}}_{P}$.

## 4. Positioning Algorithm for the PSDM Model

To address the main shortcomings of the pseudo-linearization closed-form method derived in the previous section, this section first redesigned a pseudo-linearization matrix by separating variables and reconstructing the pseudo-linearization equation, which avoids the influence of measurement noise on the elements of the matrix and reduces the number of signals required to achieve positioning. Then, the spatial constraints related to the receiver position are established through the Earth ellipsoid model, and the height of the BS is used to initialize the constraint conditions. On this basis, a constrained multi-step weighted least squares fusion positioning algorithm is proposed to solve the PSDM positioning model. This method reduces the influence of noise on positioning and improves the global convergence of the positioning process.

The proposed CMWLS method is implemented through four steps. In the first step, the positioning equations are redesigned, and auxiliary variables and position states are estimated separately by constraint conditions. In the second and third steps, the estimation result from the first step is utilized to improve the state estimation accuracy. Finally, let the state estimation obtained in the third step be used as the initial value, and an accurate state estimation can be obtained through the iterative WLS.

### 4.1. Pseudo Linearization Reconstruction

In the East-North-Up (ENU) coordinate system with the BS as a reference and assuming that the altitude of the receiver remains almost unchanged or changes very little in a short period of time, Equation (20) can be reconstructed as:

(20) $$\begin{array}{c} {\tilde{h}}_{1}=G_{xy}x_{xy}+q_{z}z_{u}^{e}+g_{1}^{bs}d_{1}^{bs}+\ldots g_{N^{bs}}^{bs}d_{N^{bs}}^{bs}+\\ g_{1}^{gnss}d_{1}^{gnss}+\ldots g_{N^{gnss}}^{gnss}d_{N^{gnss}}^{gnss}+\varepsilon_{1} \end{array}$$

where ${\tilde{h}}_{1}={\tilde{h}}_{P}$, $G_{xy}=(G_{P}^{enu}(:,1:2),G_{P}^{enu}(:,4:5),\ldots G_{P}^{enu}(:,3L-2:3L-1))$, $\left({*}_{m}:{*}_{n}\right)$ are elements from column/row ${*}_{m}$ to column/row ${*}_{n}$,$\left(:,:\right)$ represents a matrix composed of specified rows and columns, where the left side of the comma represents the rows of the original matrix and the right side represents the columns of the original matrix, the state vector is $x_{xy}=[x_{u,1},y_{u,1},x_{u,2},y_{u,2},\ldots x_{u,L},y_{u,L}{]}^{T}$,$q_{z}=G_{P}^{enu}(:,3)$,$z_{u}^{e}=z_{u,1}\approx z_{u,k}$,$g_{i}^{bs}=G_{P}^{enu}(:,3L+i)$, and $g_{j}^{gnss}=G_{P}^{enu}(:,3L+N^{bs}+j)$, $G_{P}^{enu}$ is the result of ${\tilde{G}}_{P}$ after ENU coordinate conversion, and $\varepsilon_{1}={\tilde{C}}_{P}{\tilde{\varepsilon }}_{P}$, $d_{1}^{bs}\ldots d_{N^{bs}}^{bs}$ and $d_{1}^{gnss}\ldots d_{N^{gnss}}^{gnss}$ are the distances between the signal sources and the receiver at the initial epoch, this is the auxiliary variable added to $x_{P}$ in Equation (15). However, the dimension of $G_{xy}$ in Equation (20) is $N^{gnss}N^{bs}(L-1)\times 2L$, which still cannot achieve full rank when $N^{gnss}+N^{bs}\leq 3$, $N^{gnss}\geq 1$ and $N^{bs}\geq 1$. Therefore, Equation (20) can be further redesigned as:

(21) $$\begin{array}{c} {\bar{h}}_{1}={\bar{G}}_{xy}{\bar{x}}_{xy}+q_{z}z_{u}^{e}+g_{1}^{bs}d_{1}^{bs}+\ldots g_{N^{bs}}^{bs}d_{N^{bs}}^{bs}\\ +g_{1}^{gnss}d_{1}^{gnss}+\ldots g_{N^{gnss}}^{gnss}d_{N^{gnss}}^{gnss}+\varepsilon_{1} \end{array}$$

where

$$\begin{array}{c} {\bar{h}}_{1}=\left\{\begin{array}{cc} {\tilde{h}}_{1} & N^{bs}+N^{gnss}\geq 4\\ {\tilde{h}}_{1}-G_{LL}x_{L} & else \end{array}\right.,\;{\bar{G}}_{xy}=\left\{\begin{array}{cc} G_{xy} & N^{bs}+N^{gnss}\geq 4\\ G_{xy}(:,1:2L-2) & else \end{array}\right.,\;{\bar{x}}_{xy}=\\ \left\{\begin{array}{cc} x_{xy} & N^{bs}+N^{gnss}\geq 4\\ x_{xy}(1:2(L-1),1) & else \end{array}\right.,\;G_{LL}=G_{xy}(:,2L-1:2L),\;\mathrm{and}\;x_{L}=[x_{u,L},y_{u,L}{{]}^{T}}_{2\times 1}. \end{array}$$

In Equation (21), when the total number of cellular SOP–GNSS is as $N^{gnss}+N^{bs}=3$, ${\bar{G}}_{xy}$ can maintain full rank. At the initial time, $x_{L}$ is unknown. Therefore, set $x_{L}$ as the position of a certain BS at the initial time. Then, ${\bar{x}}_{xy}$ can be estimated. Considering the slow motion of the receiver, update $x_{L}$ to ${\hat{\bar{x}}}_{xy}(2L-3:2L-2,1)$. After several iterations, the estimation can converge.

According to the principle of weighted least squares estimation, the position solution of the receiver in Equation (20) can be expressed as:

(22) $$\begin{array}{c} {\bar{x}}_{xy}=({\bar{G}}_{xy}{)}^{pinv}({\bar{h}}_{1}-q_{z}z_{u}^{e}-g_{1}^{bs}d_{1}^{bs}-\ldots g_{N^{bs}}^{bs}d_{N^{bs}}^{bs}-\\ g_{1}^{gnss}d_{1}^{gnss}-\ldots g_{N^{gnss}}^{gnss}d_{N^{gnss}}^{gnss}) \end{array}$$

where $({\bar{G}}_{xy}{)}^{pinv}=({\bar{G}}_{xy}^{T}Q_{xy}^{-1}{\bar{G}}_{xy}{)}^{-1}{\bar{G}}_{xy}^{T}Q_{xy}^{-1}$ is the pseudo-inverse of a matrix, $Q_{xy}=E(\omega_{1}\omega_{1}^{T})$, and $\omega_{1}$ is the measurement error of vector $\left({\bar{h}}_{1}-q_{z}z_{u}^{e}-g_{1}^{bs}d_{1}^{bs}-\ldots g_{N^{bs}}^{bs}d_{N^{bs}}^{bs}-g_{1}^{gnss}d_{1}^{gnss}-\ldots g_{N^{gnss}}^{gnss}d_{N^{gnss}}^{gnss}\right)$, which can be initially set as an identity matrix. It can be seen from Equation (22) that if estimates of $z_{u}^{e}$, $d_{1}^{bs}\ldots d_{N^{bs}}^{bs}$ and $d_{1}^{gnss}\ldots d_{N^{gnss}}^{gnss}$ can be obtained, then estimates of ${\bar{x}}_{xy}$ can be easily obtained.

### 4.2. Establish Spatial Constraints

The BSs and the receiver are distributed on the surface of the Earth. The height difference between the BS and the receiver is small, usually only a few tens of meters. After cellular SOPs enter, the height of the BS constrains the receiver to be on the surface of the Earth’s ellipsoid.

Assuming that in the Earth Centered Earth Fixed (ECEF) coordinate system, the position coordinates of the receiver on the Earth’s surface are $r_{u}=(x_{u},y_{u},z_{u}{)}^{T}$, the projection relationship between this coordinate and latitude-longitude-altitude in the ellipsoid model is as follows:

(23) $$\begin{array}{c} x_{u}=(M+d_{z})\cos(\theta_{lat})\cos(\theta_{lon})\\ y_{u}=(M+d_{z})\cos(\theta_{lat})\sin(\theta_{lon})\\ z_{u}=(M(1-e^{2})+d_{z})\sin(\theta_{lat}) \end{array}$$

where $\theta_{lat}$ and $\theta_{lon}$ are the geodetic latitude and longitude of the receiver, $d_{z}$ is the height (altitude) of the receiver from the ground surface, e=0.08181919084262149 is the first eccentricity, and $M=\frac{r_{d}}{\sqrt{1-e^{2}\sin^{2}(\theta_{lat})}}$, where $r_{d}\approx 6378.137\;\mathrm{km}$ is the radius of the Earth.

According to Equation (23) and the sine-cosine operation, the position of the receiver satisfies the following constraint equation:

(24) $$x_{u}^{2}+y_{u}^{2}+(M+d_{z}{)}^{2}\sin^{2}(\theta_{lat})=(M+d_{z}{)}^{2}$$

The third term on the left side of Equation (24) can be represented by the equation $z_{u}$ as:

(25) $$(M+d_{z}{)}^{2}\sin^{2}(\theta_{lat})=\frac{z_{u}^{2}(M+d_{z}{)}^{2}}{(M(1-e^{2})+d_{z}{)}^{2}}$$

According to Equation (25), Equation (24) can be reorganized as:

(26) $$r_{u}^{T}Wr_{u}=a^{2}$$

where $a=M+d_{z}$, W=diag([1,1,1/m]), $diag([{*}_{1},\ldots {*}_{n}])$ represents a diagonal matrix with $\left[{*}_{1},\ldots {*}_{n}\right]$ as the main diagonal element, and $m=(M(1-e^{2})+d_{z}{)}^{2}$.

It can be seen that the above constraints require prior knowledge of the altitude $d_{z}$ of the receiver, which is unknown. The difference between the BS height and the receiver height in urban environments is relatively small. Therefore, in the subsequent use of this constraint for positioning, the BS’s height can be approximated as the receiver height for initialization constraint conditions.

### 4.3. The Proposed CMWLS Method for PSDM Positioning

#### 4.3.1. Initial Estimation of Position Based on Spatial Constraints WLS

- Coordinate transformation

For the receiver position of the first epoch, Equation (26) can be re-expressed as:

(27) $$r_{u,1}^{T}Wr_{u,1}=a^{2}$$

According to the established constraint relationship in Section 4.2, it can be inferred that the receiver position $r_{u,1}$ in Equation (27) is in the ECEF coordinate system. However, the receiver position state in Equation (26) is in the ENU coordinate system. Therefore, a conversion between the ECEF and ENU coordinate systems is required:

(28) $$r_{u,k}^{ecef}=S^{-1}r_{u,k}^{enu}+\chi =(S^{-1}{)}_{1:3,1:2}({\bar{x}}_{xy}{)}_{2k-1:2k}+(S^{-1}{)}_{1:3,3}z_{u}^{e}+\chi$$

where $r_{u,k}^{ecef}=r_{u,k}$ is the position vector of the receiver in ECEF coordinates, $r_{u,k}^{enu}$ is the position vector of the receiver in ENU coordinates, $S=\left[\begin{array}{ccc} -\sin(\theta_{lon}) & \cos(\theta_{lon}) & 0\\ -\sin(\theta_{lat})\cos(\theta_{lon}) & -\sin(\theta_{lat})\sin(\theta_{lon}) & \cos(\theta_{lat})\\ \cos(\theta_{lat})\cos(\theta_{lon}) & -\cos(\theta_{lat})\sin(\theta_{lon}) & \sin(\theta_{lat}) \end{array}\right]$ is the ECEF-ENU coordinate transfer matrix, and χ is the coordinate vector of the reference station in the two coordinate system transformations.

- Obtain the estimation of auxiliary variables

Substituting Equations (22) and (28) into Equation (27) yields:

(29) $${\bar{M}}^{T}W\bar{M}=a^{2}$$

where $\bar{M}=(M_{xy}({\bar{h}}_{1}-q_{z}z_{u}^{e}-gd)+M_{z}z_{u}^{e}+\chi )$,$gd=g_{1}^{bs}d_{1}^{bs}+\ldots g_{N^{bs}}^{bs}d_{N^{bs}}^{bs}+g_{1}^{gnss}d_{1}^{gnss}+\ldots g_{N^{gnss}}^{gnss}d_{N^{gnss}}^{gnss}$, $M_{xy}=(S^{-1}{)}_{1:3,1:2}V(G_{xy}{)}^{pinv}$, $V=\left[\begin{array}{cccc} 1 & 0 & \ldots & 0\\ 0 & 1 & \ldots & 0 \end{array}\right]$, $M_{z}=(S^{-1}{)}_{1:3,3}$, the subscript represents the matrix composed of corresponding rows and columns.

Reorganizing Equation (29) into the form of a system of multivariate linear equations, then the $z_{u}^{e}$ can be expressed by $d_{1}^{bs}\ldots d_{N^{bs}}^{bs}$ and $d_{1}^{gnss}\ldots d_{N^{gnss}}^{gnss}$:

(30) $$z_{u}^{e}=z^{e}\left(d_{1}^{bs},\ldots ,d_{N^{bs}}^{bs},d_{1}^{gnss},\ldots ,d_{N^{gnss}}^{gnss}\right)$$

where $z^{e}(*)$ is a polynomial function.

On the other hand, substitute Equations (22) and (28) into $(d_{1}^{bs}{)}^{2}=(r_{u,1}{)}^{T}r_{u,1}-2(r_{1}^{bs}{)}^{T}r_{u,1}+(r_{1}^{bs}{)}^{T}r_{1}^{bs}$ gives:

(31) $$\begin{array}{c} (d_{1}^{bs}{)}^{2}=\\ a^{2}+\mu (z_{u}^{e}{)}^{2}-2(r_{1}^{bs}{)}^{T}(M_{xy}({\bar{h}}_{1}-q_{z}z_{u}^{e}-gd)+M_{z}z_{u}^{e}+\chi )+(r_{1}^{bs}{)}^{T}r_{1}^{bs} \end{array}$$

where $\mu =1-\left(\frac{1}{m}\right)$. Set Equation (31) as $R_{1}^{bs}$. Similarly, multiple $R_{i}^{bs}$ and $R_{j}^{gnss}$ values can be obtained by performing the above operation. Organize these equations into a system of linear equations containing variables $d_{1}^{bs}\ldots d_{N^{bs}}^{bs}$, $d_{1}^{gnss}\ldots d_{N^{gnss}}^{gnss}$ and $z_{u}^{e}$. By substituting the expression for $z_{u}^{e}$ obtained in Equation (36), a system of equations containing only the variables $d_{1}^{bs}\ldots d_{N^{bs}}^{bs}$ and $d_{1}^{gnss}\ldots d_{N^{gnss}}^{gnss}$ can be obtained. The number of equations is the same as the number of unknown variables. Therefore, the estimation ${\hat{d}}_{1}^{bs}\ldots {\hat{d}}_{N^{bs}}^{bs}$ and ${\hat{d}}_{1}^{gnss}\ldots {\hat{d}}_{N^{gnss}}^{gnss}$ can be obtained by solving a system of multivariate linear equations. Furthermore, through Equation (36), the estimation ${\hat{z}}_{u}^{e}$ of $z_{u}^{e}$ can also be obtained.

- Obtain position state estimation

Substituting ${\hat{z}}_{u}^{e}$, ${\hat{d}}_{1}^{bs}\ldots {\hat{d}}_{N^{bs}}^{bs}$ and ${\hat{d}}_{1}^{gnss}\ldots {\hat{d}}_{N^{gnss}}^{gnss}$ into Equation (22), the 2D position estimate ${\hat{\bar{x}}}_{xy}$ of the receiver can be obtained as:

(32) $$\begin{array}{c} {\hat{\bar{x}}}_{xy}=({\bar{G}}_{xy}{)}^{pinv}({\bar{h}}_{1}-q_{z}{\hat{z}}_{u}-g_{1}^{bs}{\hat{d}}_{1}^{bs}-\ldots g_{N^{bs}}^{bs}{\hat{d}}_{N^{bs}}^{bs}-\\ q_{1}^{gnss}{\hat{d}}_{1}^{bs}-\ldots q_{N^{gnss}}^{gnss}{\hat{d}}_{N^{gnss}}^{gnss}) \end{array}$$

Furthermore, the 3D position can be obtained as follows:

(33) $${\hat{x}}_{1}^{enu}=[({\hat{x}}_{xy}{)}^{T},\;{\hat{z}}_{u}^{e}{]}^{T}$$

where ${\hat{x}}_{xy}=\left\{\begin{array}{cc} \left({\hat{\bar{x}}}_{xy}\right) & N^{bs}+N^{gnss}\geq 4\\ {\left[{\left({\hat{\bar{x}}}_{xy}\right)}^{T},{\left({\hat{x}}_{L}\right)}^{T}\right]}^{T} & else \end{array}\right.$. Converting position estimation ${\hat{x}}_{1}^{enu}$ to ECEF coordinates ${\hat{\tilde{x}}}_{1}$, we can obtain: ${\hat{\tilde{x}}}_{1}=[{\hat{r}}_{u,1}^{T},\ldots ,{\hat{r}}_{u,L}^{T}{]}^{T}$.

In the constraints of Equation (27), the calculation of the parameter M requires the latitude position $\theta_{lat}$ of the receiver. However, $\theta_{lat}$ is unknown. At the initial calculation, set $M=r_{d}$. After obtaining the initial estimate of the receiver position, $\theta_{lat}$ can be calculated as:

(34) $${\hat{\theta }}_{lat}=\arctan(\frac{{\hat{z}}_{u}^{e}}{\left(||{\hat{x}}_{xy}||(1-e^{2})\right)})$$

Then, M can be updated through ${\hat{\theta }}_{lat}$. It should be noted that the proposed method still ignores high-order terms of noise, so the influence of noise cannot be completely avoided. Furthermore, if there are several sets of solutions that are similar for parameter ${\hat{d}}_{1}^{bs}\ldots {\hat{d}}_{N^{bs}}^{bs}$ and ${\hat{d}}_{1}^{gnss}\ldots {\hat{d}}_{N^{gnss}}^{gnss}$, it is difficult to select the most suitable solution. Therefore, the state estimation obtained in this step makes it difficult to achieve optimal results, so optimization of the state estimation is required.

#### 4.3.2. Elimination of Auxiliary Variables and Optimization of Initial Position Estimation

In this step, a linear equation is reconstructed according to the relationship between auxiliary variables and the initial receiver position. Performing $N_{1,i}-{Ζ}_{1,j}$ can obtain:

(35) $$\begin{array}{c} {\left(d_{i,1}^{bs}\right)}^{2}-{\left(d_{j,1}^{GNSS}\right)}^{2}-{\left(r_{i}^{bs}\right)}^{T}r_{i}^{bs}+{\left(r_{j,1}^{GNSS}\right)}^{T}\left(r_{j,1}^{GNSS}\right)=\\ -2r_{u,1}^{T}\left(r_{i}^{bs}-r_{j,1}^{GNSS}\right) \end{array}$$

Let the estimation of auxiliary variables be the measurements: ${\hat{d}}_{i,1}^{bs}={\hat{d}}_{i}^{bs}$,${\hat{d}}_{j,1}^{GNSS}={\hat{d}}_{j}^{gnss}$. By substituting it into Equation (35) and ignoring higher-order noise terms, a linear equation can be obtained as follows:

(36) $$C_{2}\varepsilon_{2}=h_{2}-G_{2}{\tilde{x}}_{2}$$

where the state vector is: ${\tilde{x}}_{2}=r_{u,1}$, the measurement vector is: $h_{2}=0.5[h_{2}^{1,1},\ldots h_{2}^{N^{bs},1},h_{2}^{2,1},\ldots h_{2}^{N^{bs},N^{gnss}}{]}^{T}$, $h_{2}^{i,j}=({\hat{d}}_{i,1}^{bs}{)}^{2}-({\hat{d}}_{j,1}^{GNSS}{)}^{2}-(r_{i}^{bs}{)}^{T}r_{i}^{bs}-(r_{j,1}^{GNSS}{)}^{T}r_{j,1}^{GNSS}$, and the linearization matrix $G_{2}$ is:

$$G_{2}=\left[\begin{array}{c} -(r_{1}^{bs}-r_{1,1}^{GNSS}{)}^{T}\\ \vdots \\ -(r_{N^{bs}}^{bs}-r_{1,1}^{GNSS}{)}^{T}\\ \vdots \\ -(r_{N^{bs}}^{bs}-r_{N^{gnss},1}^{GNSS}{)}^{T} \end{array}\right]$$

The noise vector in Equation (36) is $\varepsilon_{2}=[n_{1,1}^{bs},\ldots n_{N^{bs},1}^{bs},n_{1,1}^{gnss},\ldots n_{N^{gnss},1}^{gnss}{]}^{T}$, where $n_{i,1}^{bs}$ and $n_{j,1}^{gnss}$ are the estimation errors of the auxiliary variable in the first step. The noise matrix is as follows:

$$C_{2}=\left[\begin{array}{c} C_{1}^{gnss}\\ \vdots \\ C_{N^{gnss}}^{gnss} \end{array}\right],\;C_{j}^{bs}=\left[\begin{array}{cccccccc} d_{1,1}^{bs} & \ldots & 0 & 0 & \ldots & -d_{j,1}^{GNSS} & \ldots & 0\\ \vdots & \ddots & \vdots & \vdots & \vdots & \vdots & \vdots & \vdots \\ 0 & \ldots & d_{N^{bs},1}^{bs} & 0 & \ldots & -d_{j,1}^{GNSS} & \ldots & 0 \end{array}\right].$$

According to the WLS method, the estimation of ${\tilde{x}}_{2}$ is as follows:

(37) $${\hat{\tilde{x}}}_{2}=({G_{2}}^{T}{Q_{2}}^{-1}G_{2}{)}^{-1}G_{2}{Q_{2}}^{-1}h_{2}$$

where $Q_{2}=E((C_{2}\varepsilon_{2})(C_{2}\varepsilon_{2}{)}^{T})=C_{2}Q_{1}{C_{2}}^{T}$.

#### 4.3.3. Optimization of Multi-Epoch Position Estimation

According ${\hat{\tilde{x}}}_{1}$ and ${\hat{\tilde{x}}}_{2}$, the new linear equation is constructed as:

(38) $$C_{3}\varepsilon_{3}=h_{3}-G_{3}{\tilde{x}}_{3}$$

where the measurement vector is: $h_{3}=[({\hat{\tilde{x}}}_{2}{)}^{T},({\hat{r}}_{u,2}-{\hat{r}}_{u,1}{)}^{T},\ldots ({\hat{r}}_{u,L}-{\hat{r}}_{u,1}{)}^{T}{]}^{T}$, the state vector is: ${\tilde{x}}_{3}=[r_{u,1}^{T},r_{u,2}^{T}\ldots r_{u,L}^{T}{]}^{T}$, $G_{3}=C_{3}=\left[\begin{array}{cccc} I_{3\times 3} & 0 & 0 & 0\\ -I_{3\times 3} & I_{3\times 3} & \ldots & 0\\ \vdots & \vdots & \ddots & \vdots \\ -I_{3\times 3} & 0 & \ldots & I_{3\times 3} \end{array}\right]$, and the noise vector is $\varepsilon_{3}=[\varphi^{T},n_{2}^{T},n_{3}^{T}\ldots n_{L}^{T}{]}^{T}$, where φ is the estimation error of ${\hat{\tilde{x}}}_{2}$, and $[n_{2}^{T},n_{3}^{T}\ldots n_{L}^{T}{]}^{T}$ is the estimation error of ${\hat{\tilde{x}}}_{1}$.

According to the WLS method, the solution of Equation (38) is as follows:

(39) $${\hat{\tilde{x}}}_{3}=({G_{3}}^{T}{Q_{3}}^{-1}G_{3}{)}^{-1}G_{3}{Q_{3}}^{-1}h_{3}$$

where $Q_{3}=E((C_{3}\varepsilon_{3})(C_{3}\varepsilon_{3}{)}^{T})$. The estimation of the position in the third step of the CMWLS method can be obtained as: ${\hat{x}}^{CMWLS-3}={\hat{\tilde{x}}}_{3}$.

#### 4.3.4. Secondary Optimization of Multi-Epoch Position Estimation

Due to neglecting the high-order terms of noise up to steps, the state estimation obtained by Equation (39) is still sensitive to noise. To further reduce the impact of noise and improve the global convergence of the positioning model, the positioning result obtained in Equation (39) is used as the initial Taylor expansion value, and the nonlinear function f(x) in Equation (10) is linearized. Then, according to the WLS theory, the Gaussian Newton solution of Equation (10) is further obtained through iterative calculation. After Taylor linearization, Equation (10) can be rewritten as:

(40) $$\underset{x}{min}\frac{1}{2}(F\cdot \delta x-\tilde{m}{)}^{T}Q^{-1}(F\cdot \delta x-\tilde{m})$$

where δx is the change in state estimation, $\tilde{m}=\tilde{z}-f(x)$, and $F=\left[\begin{array}{c} F^{BS}\\ F^{GNSS} \end{array}\right]$ is the Jacobian matrix of f(x), where

$$\begin{array}{c} F^{GNSS}=\left[\begin{array}{c} F_{1}^{GNSS}\\ \vdots \\ F_{N^{gnss}}^{GNSS} \end{array}\right],\;F^{BS}=\left[\begin{array}{c} F_{1}^{BS}\\ \vdots \\ F_{N^{bs}}^{BS} \end{array}\right],\;F_{j}^{GNSS}={\left[\begin{array}{cccccc} -e_{j,1}^{gnss} & e_{j,2}^{gnss} & \ldots & 0 & e_{2}^{\delta t} & e_{2}^{\dot{\delta }}\\ \vdots & \vdots & \ddots & \vdots & \vdots & \vdots \\ -e_{j,1}^{gnss} & 0 & \ldots & e_{j,L}^{gnss} & e_{L}^{\delta t} & e_{L}^{\dot{\delta }} \end{array}\right]}_{\left(L-1)\times (3L+2\right)},\\ F_{i}^{BS}={\left[\begin{array}{ccccccc} -e_{i,1}^{bs} & e_{i,2}^{bs} & 0 & \ldots & 0 & 0 & T\\ \vdots & \vdots & \ddots & \vdots & \vdots & \vdots & \vdots \\ -e_{i,1}^{bs} & 0 & 0 & \ldots & e_{i,L}^{bs} & 0 & (L-1)T \end{array}\right]}_{\left(L-1)\times (3L+2\right)},\;e_{i,k}^{bs}=-\frac{(r_{i}^{bs}-r_{u,k}{)}^{T}}{\left||r_{u,k}-r_{i}^{bs}|\right|},\;\\ e_{j,k}^{gnss}=-\frac{(r_{j,k}^{GNSS}-r_{u,k}{)}^{T}}{\left||r_{u,k}-r_{j,k}^{GNSS}|\right|},\;e_{k}^{\delta t}=-\frac{\left(v_{j,k}^{GNSS}{)}^{T}(r_{j,k}^{GNSS}-r_{u,k}\right)}{c||r_{u,k}-r_{j,k}^{GNSS}||},\;e_{k}^{\dot{\delta }}=-\frac{\left(k-1)T(v_{j,k}^{GNSS}{)}^{T}(r_{j,k}^{GNSS}-r_{u,k}\right)}{c||r_{u,k}-r_{j,k}^{GNSS}||}+(k-1)T.\; \end{array}$$

According to the principle of weighted least squares, the Gaussian Newton solution of Equation (40) is as follows:

(41) $$\delta \hat{x}=(F^{T}Q^{-1}F{)}^{-1}FQ^{-1}\tilde{m}$$

After continuous iteration, a more accurate state estimate can be obtained as:

(42) $${\hat{x}}_{n}={\hat{x}}_{n-1}+\delta {\hat{x}}_{n}$$

where ${\hat{x}}_{n}$ is the state estimation at the n-th iteration, and the initial state is ${\hat{x}}_{0}={\hat{x}}^{CMWLS\text{-}3}$.

**Figure 2:**
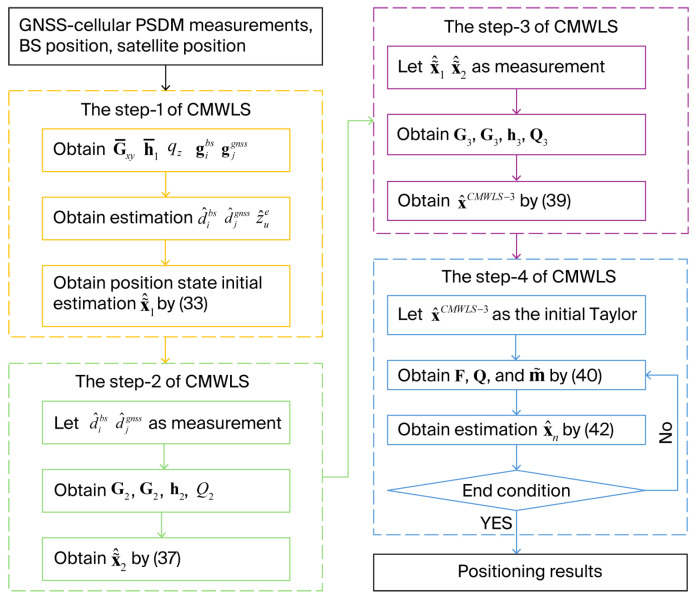
The flowchart of the proposed algorithm.

According to the content in the previous section, the detailed algorithm implementation process is shown in Figure 2 below:

Step (1). Set initial $M=r_{d}$. Calculate ${\bar{G}}_{xy},\;{\bar{h}}_{1},\;q_{z},\;g_{i}^{bs},\;g_{j}^{gnss}$, the estimate of $d_{1}^{bs},\ldots d_{N^{bs}}^{bs}$ and $d_{1}^{gnss},\ldots d_{N^{gnss}}^{gnss}$ can be obtained by Equation (31), and then the ${\hat{z}}_{u}^{e}$ can be obtained by Equation (30). Furthermore, the estimate of the initial receiver position ${\hat{\tilde{x}}}_{1}$ with L epoch can be obtained through Equation (33).

Step (2). Let ${\hat{d}}_{i,1}^{bs}={\hat{d}}_{i}^{bs}$, and ${\hat{d}}_{j,1}^{GNSS}={\hat{d}}_{j}^{gnss}$ and calculate $G_{2},\;Q_{2},\;C_{2},\;h_{2}$. Auxiliary variables are eliminated and the position estimate of the first epoch can be obtained by Equation (37).

Step (3). Calculate $G_{3},\;Q_{3},\;C_{3},\;h_{3}$. Step 3 of CMWLS method can be executed. The optimized position estimation the ${\hat{x}}^{CMWLS-3}$ can be obtained through Equation (39).

Step (4). Set the maximum number of iterations. The position estimate ${\hat{x}}^{CMWLS-3}$ is as the initial Taylor expansion value of the PSDM model. Via iterative calculations, the state estimation $\hat{x}={\hat{x}}_{n+1}$ can be obtained via Equation (42).

## 5. Performance Analysis

### 5.1. Convergence Analysis of Positioning

The proposed CMWLS method utilizes the results of steps 1 to 3 to initialize the PSDM positioning model based on Taylor series linearization. Steps 1 to 3 of CMWLS are defined as a constrained three-step weighted least squares (CTWLS) method. As mentioned earlier, large initialization errors have a significant impact on the PSDM model based on Taylor linearization, which may make it difficult for positioning to converge to the global optimum. Therefore, to better analyze the convergence of the different methods for initializing the PSDM positioning model, a simulation experiments were conducted.

**Figure 3:**
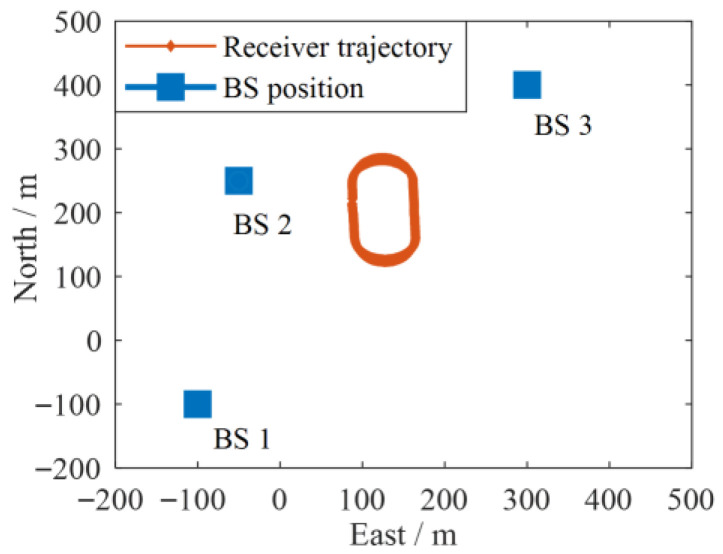
Simulation experiment environment.

Three BSs and three GNSS satellites were deployed in the simulation experiment. Information regarding the BSs and GNSS satellites is shown in Table 1. To increase the credibility of the simulation experiment, the satellite position was obtained from the real GPS data collected from the field test. The skyplot information of the GPS satellite is shown in next Section. The simulated trajectory of the receiver is shown in Figure 3. The altitude of the receiver is set to 53 m. Equidistant measurements at intervals of 1 s are extracted from the simulated trajectory, with 1 s as one epoch. A total of 200 epochs of GNSS and BS pseudorange measurements were obtained for testing and verification.

According to the number of visible signals, the experiment can be divided into six scenarios: 3 GSs-2 BSs, 2 GSs-3 BSs, 2 GSs-2 BSs, 2 GSs-1BS, 1GS-1BS, and 3 BSs. In GNSS and BS pseudorange measurements, zero-mean Gaussian white noises with standard deviations (STD) of 3 m and 2 m are added, respectively. The receiver’s initial clock bias is 5 m, and the clock offset is 0.1 m/s. The clock biases of three BSs are 5 m, 15 m, and 25 m, respectively.

For the PSDM positioning model based on Taylor linearization, a comparison of the convergence of the two methods, random initialization and CTWLS initialization, was conducted. The positioning results obtained in steps 1 to 3 of the proposed method can be used as the initial value for Taylor linearization. Therefore, steps 1 to 3 of the proposed algorithm are defined as the CTWLS method. The random initialization value was randomly selected within the 1000 m coverage range of BS 1. A threshold was set to determine the convergence of the positioning results.

**Figure 4:**
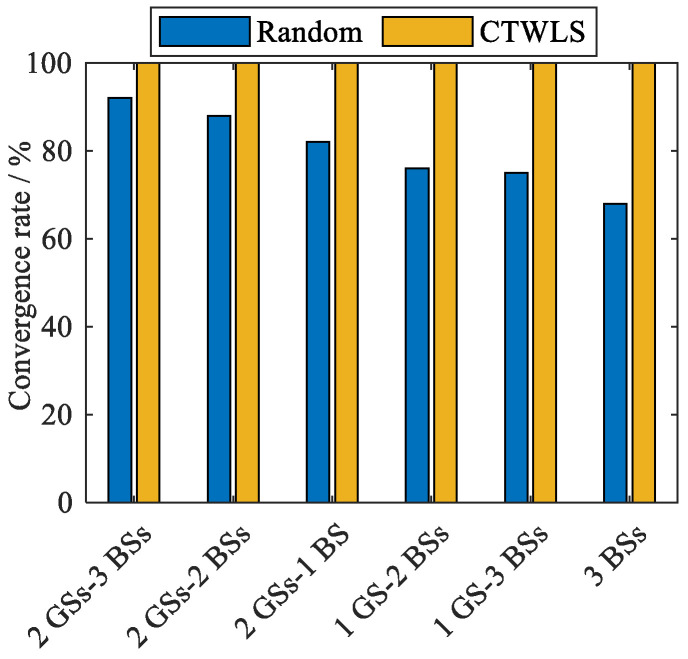
Convergence of the PSDM model for different initialization methods.

The convergence of the PSDM positioning model solved by two different initialization methods for the six cases is shown in Figure 4. From the results in the figure, it can be seen that (1) compared to random initialization methods, the proposed method has better global convergence, and (2) after fusing with fewer GNSS signals, random initialization still has the problem of not converging to the optimal results.

### 5.2. Positioning Accuracy Analysis

In this section, the positioning estimation error of the proposed CMWLS method is derived, and then the positioning performance of different pseudo-linearization methods for the PSDM positioning is analyzed through simulation experiments.

#### 5.2.1. Theoretical Analysis

For the PSDM positioning model, matrix F is not affected by noise. Therefore, the state estimation and measurement vector containing error terms can be re-expressed as:

(43) $$\hat{x}=x+\Delta x,\;\tilde{m}=m+\Delta m$$

where x and m are true value, Δx and Δm are error. According to the WLS estimation, it can be concluded that:

(44) $$x+\Delta x={\left(F^{T}Q^{-1}F\right)}^{-1}F^{T}Q^{-1}\left(m+\Delta m\right)$$

The true estimation of the state is $x=(F^{T}Q^{-1}F{)}^{-1}F^{T}Q^{-1}h$. Therefore:

(45) $$\Delta x=(F^{T}Q^{-1}F{)}^{-1}F^{T}Q^{-1}\Delta m$$

Since Δm=Δε, the covariance matrix of state estimation of the PSDM model is:

(46) $$E((\Delta x)(\Delta x{)}^{T})=(F^{T}Q^{-1}F{)}^{-1}$$

According to the definition of the Fisher information matrix, the information matrix $I_{GNSS-BSs}^{PSDM}$ of PSDM measurements is:

(47) $$I_{GNSS-BSs}^{PSDM}=-E\left(\frac{𝜕\ln f(\Delta \tilde{\rho },\Delta \rho )}{𝜕\Delta \rho^{bs}𝜕\Delta \rho^{GNSS}}\right)=Q^{-1}$$

where ln is the log function and $f(\tilde{\rho },\rho )$ is the probability density function. Furthermore, for Equation (40), the state estimation CRLB is:

(48) $${\hat{x}}_{CRLB}=diag\left({\left(F^{T}I_{GNSS-BSs}^{PSDM}F\right)}^{-1}\right)$$

According to Equations (46) and (48), the positioning accuracy of the proposed method can reach the positioning CRLB.

#### 5.2.2. Simulation

To better verify the differences in positioning performance of different positioning algorithms for PSDM models, a simulation was conducted with 2 GNSS satellites (GS) and 2 BSs. We selected numbers 1 and 2 from Table 1 for the satellites and BSs, respectively. The positioning performance of different algorithms is compared using root mean square error (RMSE), with the standard deviation of measurement noise set as the variable.

**Figure 5:**
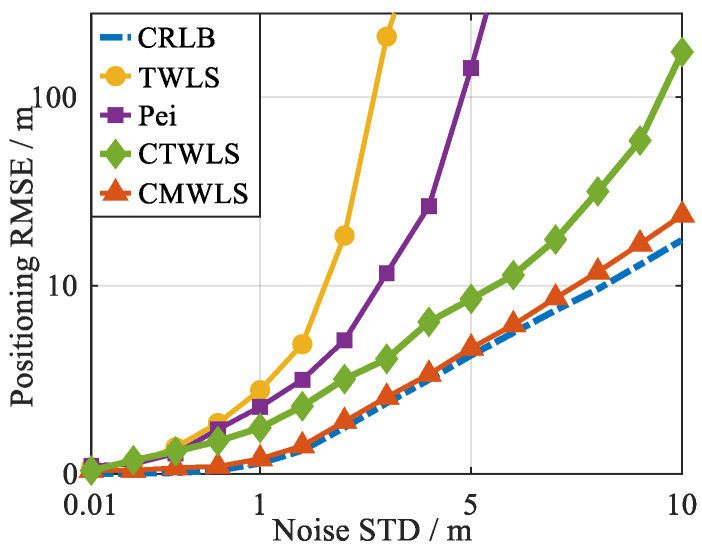
Comparison of positioning errors of different positioning methods.

The method proposed by Pei et al. in [37] is a state-of-the-art study in utilizing the constraints of the Earth ellipsoid model for positioning. In addition, steps 1 to 3 of our proposed method provide the closed-form solution of pseudo-linearization, and the positioning results of CTWLS can be used as the initial value for Taylor linearization. Therefore, in the case of 2 GSs-2 BSs, the performance of the proposed method will be compared with the TWLS, Pei, and CTWLS methods. The 2D positioning RMSEs of the four methods under different levels of noise are shown in Figure 5, and the result is an average of 200 simulations. The quantified results of the positioning errors presented in Figure 5 are shown in Table 2.

From the results in Figure 5 and Table 2, it can be seen that compared to other methods, the proposed CMWLS algorithm has better positioning performance, and the positioning accuracy is closer to the theoretical error lower bound than other methods. Compared to the TWLS and Pei methods, the CTWLS method has better performance and is less affected by noise, which proves the effectiveness of pseudo-linearization reconstruction and the CTWLS method. Overall, under the same noise conditions, the proposed CMWLS algorithm exhibits better positioning performance and lower noise sensitivity compared to the other positioning methods.

### 5.3. Analysis of the Influence of Signal Number and Epoch Number on Positioning

According to the structure of the Jacobian matrix F in the PSDM model, the rank of F satisfies: $rank(F)\leq \min((N^{bs}+N^{gnss})(L-1),p)$, where p is the number of states. Therefore, the condition for the PSDM model to have non-zero solutions is: $rank\left(F\right)=p$. For 2D positioning, p=2L+2, the following conditions should be met:

(49) $$(N^{bs}+N^{gnss})(L-1)\geq 2L+2$$

Based on the values of $\left(N^{bs}+N^{gnss}\right)$ and L, it can be determined whether matrix F is full rank. For example, when $(N^{bs}+N^{gnss})\geq 3$ and L≥5, the 2D positioning can be achieved. When $(N^{bs}+N^{gnss})\leq 2$ and the receiver is dynamic, no matter how many epoch observations are collected, 2D positioning cannot be achieved.

Next, based on Equation (49), the minimum number of observation epochs satisfies:

(50) $$L\geq \frac{2+(N^{bs}+N^{gnss})}{N^{bs}+N^{gnss}-2}$$

**Figure 6:**
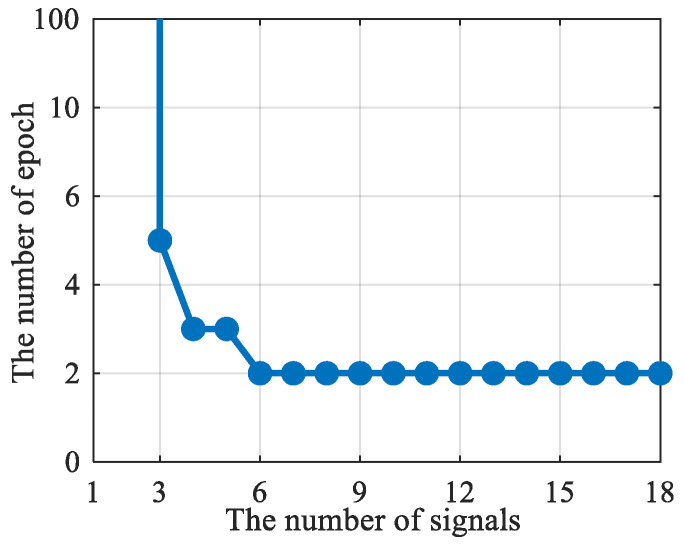
The relationship between the number of visible signals required for PSDM positioning and the number of observation epochs.

The relationship between the number of visible signals and the minimum required number of epochs is shown in Figure 6. It can be seen that as the number of visible signals increases, the minimum number of epochs required decreases.

Based on the above analysis, compared to TDOA or TOA positioning, the proposed PSDM model and method can achieve positioning in low observable environments where both visible GNSS signals and cellular SOPs are insufficient.

**Figure 7:**
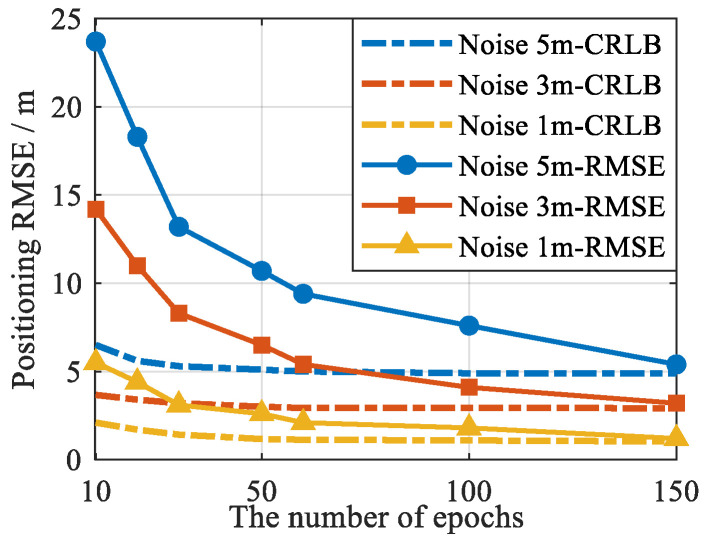
The relationship between the number of visible signals required for PSDM positioning and the number of epochs.

In addition, in the PSDM model, the number of measurements is related to the number of signals and epochs. Usually, increasing the number of visible signals can help improve the performance of positioning. To verify the impact of different numbers of epochs on the positioning performance of the proposed model and method, a simulation was conducted. In the experiment, pseudorange measurements were extracted from 300 s of data at different time intervals, resulting in different numbers of epochs. The cellular SOP–GNSS pseudorange measurements have added Gaussian noise with the same STD, the STDs are 5 m, 3 m, and 1 m. The positioning RMSEs of the proposed method under different numbers of epochs are shown in Figure 7. It can be seen from the figure that increasing the number of epochs effectively improves the positioning accuracy of the PSDM positioning model and reduces the lower bound of the theoretical error.

### 5.4. Analysis of the Impact of Receiver’s Clock Drift Estimation Error on Positioning Accuracy

In this paper, the clock drift estimation of the receiver is obtained by keeping the receiver stationary, collecting the cellular signal pseudorange measurements, and applying linear fitting. GNSS signals are not used to estimate the receiver’s clock drift. In an urban environment, cellular signals are affected by multipath and noise. However, the cellular BS is static. When the receiver is also stationary, the multipath environment between the receiver and the BS is almost unchanged, which means the multipath effect for cellular signals changes little or not at all over time. In other words, when the receiver is stationary, the effect of multipath on the pseudorange measurements of cellular signals in different epochs can be regarded as almost constant. Therefore, considering the stability of the BS clock bias in a short time, although the cellular signal is affected by the multipath effect in urban areas, the variation in the pseudorange observations of the cellular signal at different times when the receiver is stationary can be considered as being caused by the receiver clock bias and noise. The receiver’s clock drift estimation accuracy is mainly affected by noise rather than the multipath effect.

**Figure 8:**
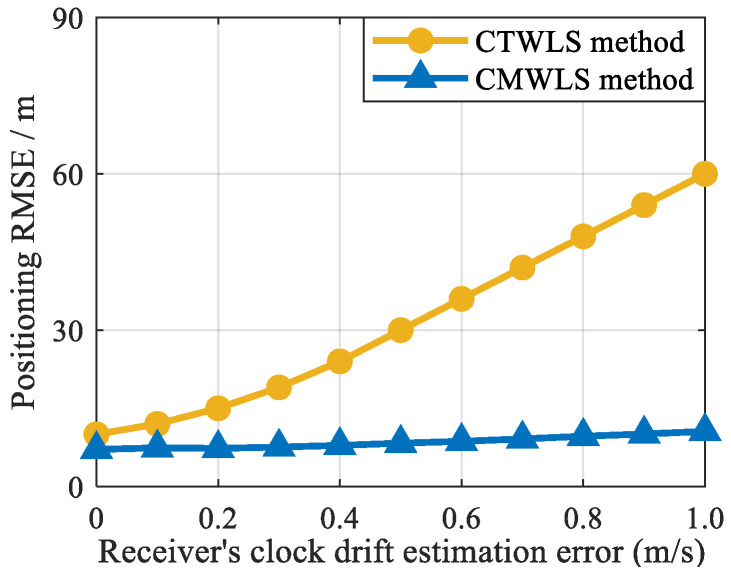
Positioning accuracy under different receiver’s clock drift estimation errors.

To better analyze the sensitivity of the receiver’s clock drift estimation error to positioning, a simulation was designed. Two GNSS satellites and two BSs were used for positioning in the simulation. Gaussian noise with a standard deviation of 5 m is added to the GNSS and cellular signal pseudorange measurements. Set the initial clock error to 5 m, the clock drift is 1 m/s, and the clock drift estimation error to 0.1–1 m/s at an interval of 0.1 m/s. The impact of the estimation error of the receiver‘s clock drift on the positioning accuracy is shown in Figure 8. The results show that the positioning error of the CTWLS and the proposed CMWLS methods increases as the receiver’s clock drift estimation error increases. However, although the receiver clock drift estimation error has some impact on the positioning results of CTWLS, the positioning accuracy of CTWLS remains in the order of tens of meters. Faced with large receiver’s clock drift estimation errors, the final positioning accuracy of the proposed CMWLS method decreases by only 2–3 m, and the impact on final positioning accuracy is relatively small. These results indicate that although the receiver’s clock drift estimation error affects the positioning accuracy of the CTWLS method (steps 1 to 3 of the CMWLS), it has little impact on the final positioning accuracy of the proposed CMWLS method in this paper. In general, the final positioning accuracy of the proposed method is less sensitive to the receiver’s clock drift estimation error.

### 5.5. Analysis of the Computational Efficiency and Real-Time Feasibility of the Algorithm

The CMWLS method proposed in this paper includes multi-step calculations and iterations. The traditional Taylor series expansion iterative method also obtains the positioning solution through iteration. To compare the computational efficiency and real-time feasibility of the proposed CMWLS algorithm and the traditional Taylor iteration method, a simulation experiment is conducted with two satellites and two BSs. Gaussian noise with a standard deviation of 5 m is added to the pseudorange measurements of the two signals. The initial clock bias is set to 5 m, and the clock drift is set to 1 m/s. In the traditional Taylor series expansion method, the initial value of the receiver position is set to the position of BS 1.

**Figure 9:**
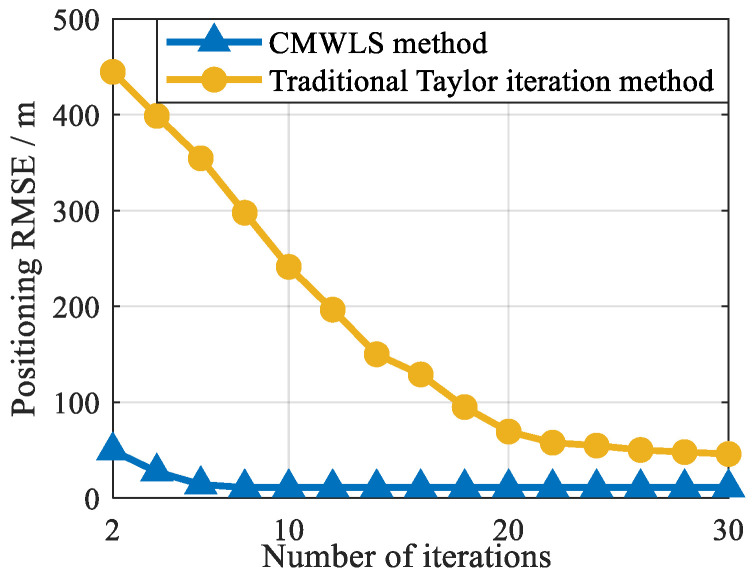
The number of iterations required by the proposed algorithm and the traditional Taylor series expansion iterative method to achieve positioning convergence.

The CMWLS method and the traditional Taylor series expansion iterative method require iteration, and the iteration will increase computational complexity. Therefore, the computational complexity of the two algorithms can be compared using the number of iterations required for iterative convergence. The number of iterations required by both the proposed algorithm and the traditional Taylor series expansion iterative method to achieve positioning convergence are shown in Figure 9. According to the experimental results in Figure 9, in the absence of exact information regarding the initial receiver position, the proposed CMWLS algorithm requires fewer iterations to achieve positioning convergence; only 4–5 iterations are required for convergence. However, the traditional Taylor series expansion iterative method requires more iterations to achieve convergence, about 15–20 times. Therefore, the proposed CMWLS algorithm has better convergence and lower computational complexity than the traditional Taylor series expansion iterative method.

**Figure 10:**
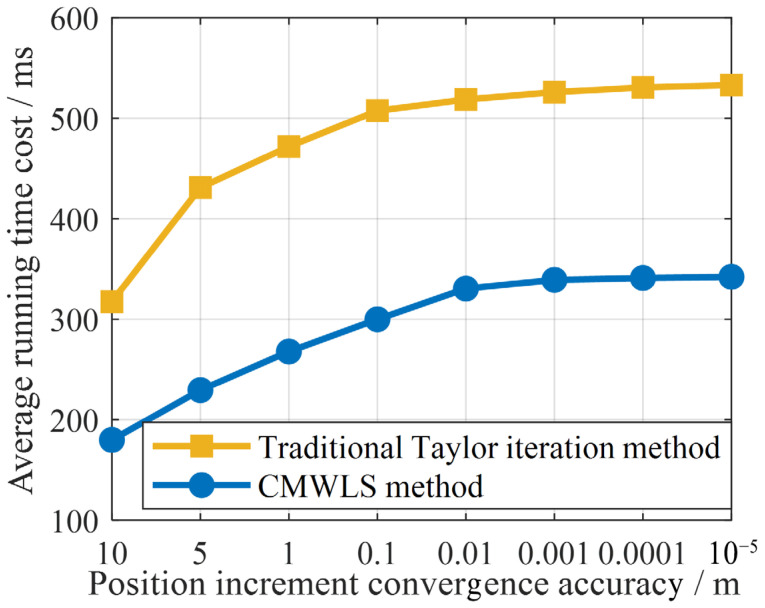
The average running time cost of the CMWLS algorithm and the traditional Taylor iterative algorithm at different convergence accuracies.

In addition, the time cost of the algorithm is also an important index for evaluating the computational efficiency and real-time feasibility. Let the accuracy of the 3D position state increment of the receiver be taken as the threshold condition at the end of the iteration process. The number of iterations required for different algorithms to converge to the same threshold accuracy varies, resulting in different running time costs of the algorithms. Moreover, the average running time cost of the algorithm can directly reflect the calculation efficiency and real-time feasibility. The average running time costs of the two algorithms at different convergence accuracies are shown in Figure 10. It can be seen from the results that compared with the traditional Taylor series expansion iterative method, the proposed CMWLS method takes a significantly shorter time to converge; this is associated with the lower number of iterations.

In summary, the experimental results in Figure 9 and Figure 10 prove that the computational efficiency and real-time feasibility of the proposed CMWLS algorithm in solving the PSDM model are higher than those of the traditional Taylor series expansion iterative method. This suggests that the proposed method is more suitable for real-time application in real-world environments.

### 5.6. Analysis of the Effect of BS-Receiver Distance on the Validity of the Height Approximation Constraint

In the proposed CMWLS method, the BS height is used to approximate the receiver’s height for initialization constraints and achieving positioning. Under the same height difference, the different distances between the receiver and the BS will lead to different projection errors. The effectiveness of the BS height approximation constraint is therefore related to the relative position between the receiver and the BS. The different pseudorange modeling errors caused by different projection errors may affect the positioning accuracy somewhat, thus affecting the effectiveness of this height approximation constraint.

**Figure 11:**
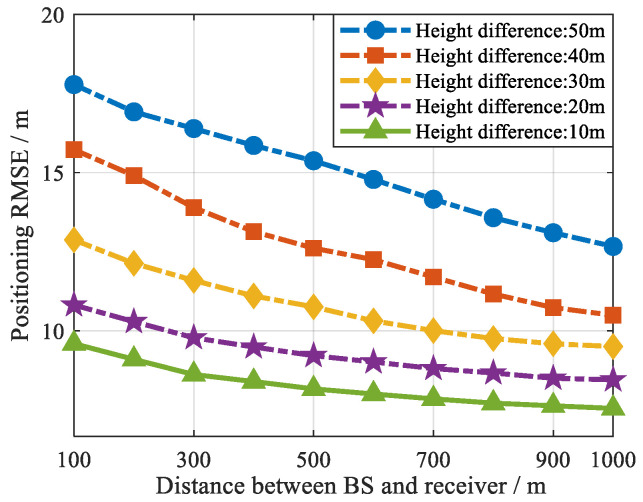
Relationship between the effectiveness of the height approximate constraints and the distance of the receiver-BS.

To better analyze the effectiveness and robustness of using the BS height to approximate the receiver height to initialize constraints, a simulation experiment was designed. The simulation was carried out with two satellites and two BSs. Set the distance between BS and the receiver as a variable. Gaussian noise with a standard deviation of 5 m was added to the satellite signal and the BS signal pseudorange measurements. Since the effective coverage range of cellular signals used for opportunistic positioning is usually within 1 km, we mainly consider BSs within 1 km. The relationship between the distance from the BS and the receiver and the effectiveness of the approximate height constraint is shown in Figure 11. The index of effectiveness is displayed through the final positioning RMSE.

According to the experimental results in Figure 11, it can be seen that the distance between the BS and the receiver affects the effectiveness of using the BS height to approximate the receiver height to initialize the constraint to a certain extent. Under the same BS height approximation, the farther the BS is from the receiver, the lower the positioning RMSE after this height approximation constraint. This is because the larger the distance between the BS and the receiver, the lower the elevation between the cellular signal and the receiver, and the smaller the proportion of pseudorange modeling errors or projection errors in real pseudorange measurements.

More importantly, according to the results from Figure 11, although the distance between the BS and the receiver is related to the effectiveness of the height approximation constraint, it has little impact on effectiveness and the final positioning error. In Figure 11, under the same height difference, after initializing the constraint by using the height of the BS when 1 km away and 100 m away, respectively, the final positioning accuracy difference of the proposed method is only within 5 m. Therefore, the experimental results prove that using BS height to approximate the receiver’s height to initialize the constraint has strong robustness. Under the same BS height approximation, the different distance between the BS and the receiver has a small impact on the effectiveness of this approximation and final positioning accuracy.

## 6. Experimental Results and Analysis

To evaluate the performance of the proposed model algorithm, the field tests were conducted. This section first introduces the experimental equipment and the test environment. Then, experimental results were shown. Specifically, the performance of the proposed method was compared and analyzed with other state-of-the-art methods. Overall, the performance of the proposed positioning method in low-observability environments was comprehensively validated.

### 6.1. Experimental Equipment

The experimental platform was built to collect and process GNSS–cellular signals. This platform uses a Y590s version software-defined radio (SDR) receiver produced by V3 technology company in Beijing, China, which is based on ADRV9009 produced by Analog Devices, Inc (ADI) company in Norwood, MA, USA.

The hardware setup of the platform includes the following:

(1). Trimble antennas for collecting GNSS signals and consumer-grade cellular omnidirectional antennas for collecting cellular signals. (2). The RF front-end of the SDR receiver integrates dual ADRV9009 produced by Analog Devices, Inc (ADI) company in Norwood, MA, USA and an FPGA of ZYNQ 7100 produced by Xilinx company in San Jose, CA, USA, with the same clock source. (3). The OEM628E of NovAtel can obtain ground-truth results using GNSS signals. (4). PCIe cable, storage and battery, etc.

The software setup of the platform consists of the following:

(1) Different GNSS and cellular SOPs software receivers designed by the Beihang communication navigation timing laboratory were used to process the collected signals on the PC to extract cellular and GNSS signal measurements. (2) The fusion positioning algorithm was validated.

**Figure 12:**
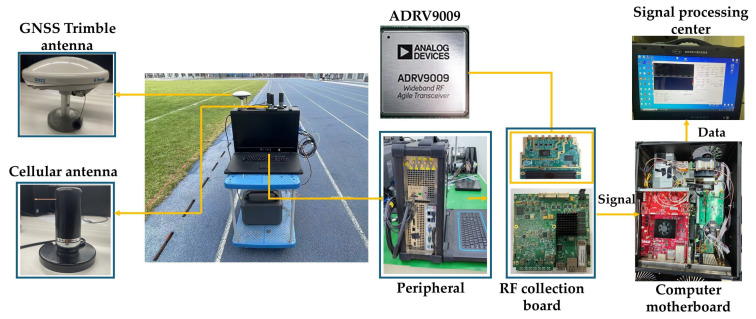
Signal collection and processing platform.

The devices and the workflow of the platform are shown in Figure 12. The RF front-end moved the received signal frequency to the baseband through the down-converter device, achieving a signal collection and quantification at the 30.72 MHz sampling rate. Then, the digital signals are stored in the PC and the signal processing is performed by different software receivers on the PC. From this, the demodulated satellite ephemeris and pseudorange measurements were obtained. Finally, the fusion positioning using the cellular SOP–GNSS was achieved by the proposed algorithms. The second version cellular SOPs software receiver was independently developed by the communication and navigation timing (CNT) laboratory of Beihang University. It can be used on software platforms, such as MATLAB produced by MathWorks company in Natick, MA, USA or Visual Studio with C++ produced by Microsoft corporation in Redmond, WA, USA, to achieve the synchronization, demodulation, and processing of cellular signals. Under conditions of sufficient cellular signals and good quality of pseudorange observations, the cellular SOPs software receiver design by the CNT laboratory of Beihang University in Beijing, China, can achieve a 2D positioning accuracy of 5–10 m using ranging observations. Our team has previously utilized the cellular SOPs software receiver to produce some valuable academic achievements, such as those in [25].

### 6.2. Experimental Environment

To better validate the performance of the proposed model and algorithm, two real signal experiments were conducted. In Experiment 1, the adequate GNSS satellites and Long Term Evolution (LTE) BS signals were collected. There are two reasons for this experimental setup. Firstly, sufficient GNSS signals ensure that the actual position coordinates of the test trajectory can be accurately obtained through OEM268E, which provides a more accurate reference for subsequent quantitative analysis of positioning errors. Secondly, different numbers of cellular SOP–GNSS signals can be selected to effectively analyze the positioning performance of the proposed positioning method. On the other hand, to better verify the performance of the proposed method in real urban occlusion scenes, a real occlusion scene (Experiment 2) was also set up.

#### 6.2.1. The Real Signal: Experiment 1

**Figure 13:**
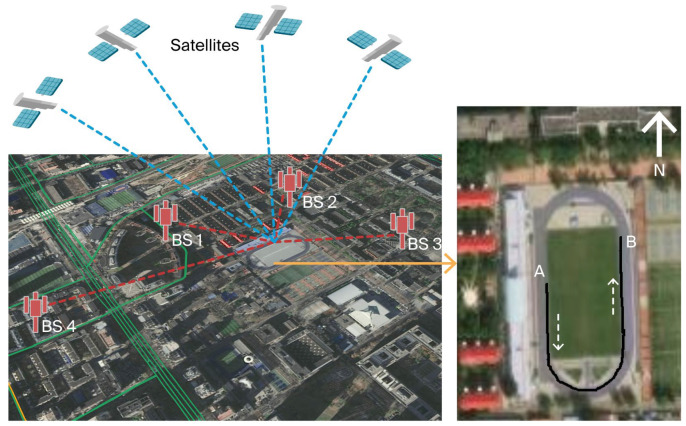
Test environment 1. The left figure shows a schematic of the experimental testing scenario. The test trajectory is shown in the figure on the right. Point A is the starting point of the receiver, point B is the ending point of the receiver’s trajectory, the black line is the receiver’s travel trajectory, the arrow is the receiver’s travel direction, and letter N and the white solid arrow are the north direction.

The data are collected and processed with the movement of the receiver, and the collection location was the campus playground of Beihang University. The equipment was placed on the experimental cart, as shown in Figure 13. The experimental environment is shown in Figure 13, where the black line is the test trajectory. The equipment is moved from point A to B. During the testing, the height of the receiver remains almost unchanged.

**Figure 14:**
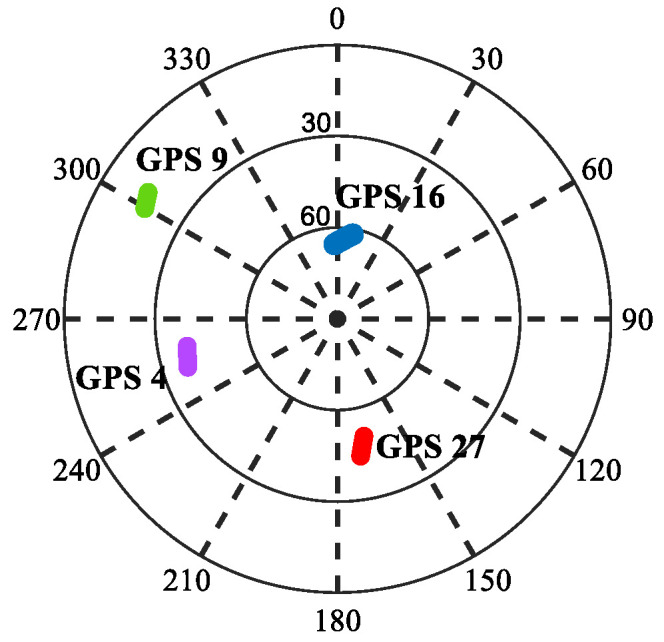
GPS satellite skyplot.

**Figure 15:**
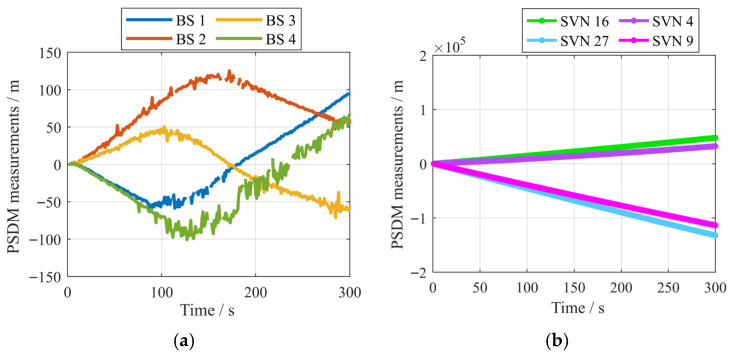
PSDM measurements of LTE BS signals and GNSS signals. (**a**) LTE BS signal PSDM measurements; (**b**) GNSS signals PSDM measurements.

In the real Experiment 1, the data collection process lasted for 302 s, and the period from 0 to 300 s was used to test the performance of the proposed algorithm. During the testing, signals from 5 GPS satellites with a frequency of 1575.42 MHz and 4 LTE BSs were collected. The skyplot of GPS satellites is shown in Figure 14. The information on LTE BS is shown in Table 3. Note that the heights of the BSs are altitudes. The PSDM measurements of LTE BS signals and GPS signals during the collection process are shown in Figure 15. The extraction interval for measurement is 1 s. The number of epochs L is an integer multiple of 1 s. The missing part in Figure 15a represents the epoch where pseudorange measurements of the LTE BSs are not available. In these epochs, the pseudorange observations of the corresponding LTE BS signals do not participate in positioning due to significant errors caused by occlusion or the multipath effect.

#### 6.2.2. The Real Signal: Experiment 2

**Figure 16:**
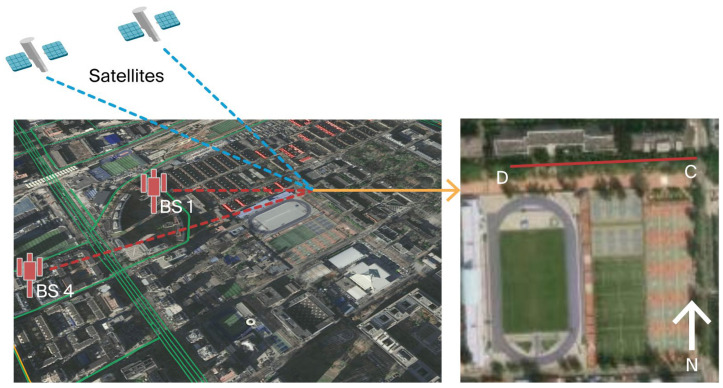
Real occlusion environment for Experiment 2. The orange arrow indicates enlargement. In the figure on the right, point D is the starting point of the receiver, point C is the ending point of the receiver’s trajectory, the red line is the receiver’s travel trajectory, and letter N and the white arrow are the north direction.

To better verify the performance of the proposed system in real low-observability environments, a real occlusion experiment was conducted on campus south road at Beihang University, as shown in Figure 16. The collection device was pushed from point C, moving from west to east, and ended at point D. The data collection period lasted for 276 s. The test route was only approximately 9.5 m away from the tall building in the north direction, while trees on the roadside obstructed the view of the zenith. Thus, there were almost no line-of-sight (LOS) signals received from the north and overhead. During the testing period of Experiment 2, the GPS signals of SVN 15, 19, 26, 27, and 30 were received with the L1 band. According to the carrier to carrier-to-noise ratio of the received GPS signals, SVN 19, 26 and 30 were considered non-LOS (NLOS) signals and should be excluded for positioning.

Similarly, due to occlusion, only BSs 1 and 4 were able to extract available measurements for most of the testing time of experiment 2. The total number of visible signals did not exceed four for more than 85% of the testing period.

#### 6.2.3. The Densely Built Areas: Experiment 3

**Figure 17:**
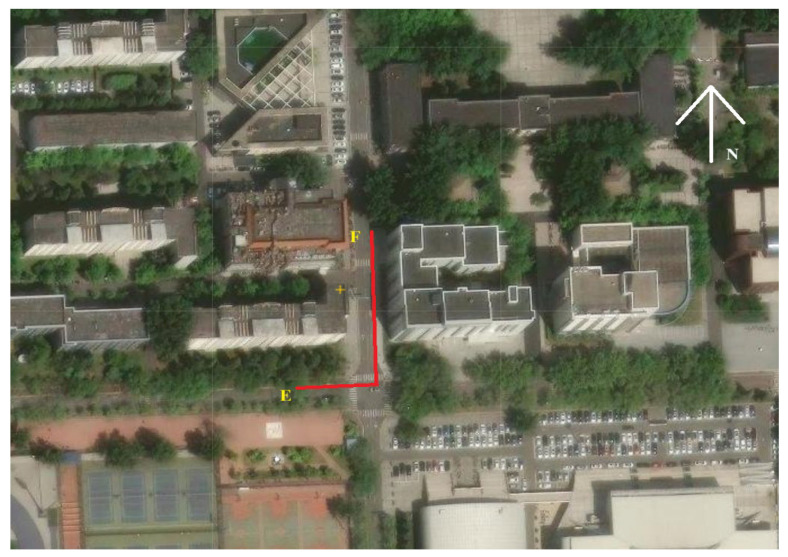
Real occlusion environment for Experiment 3. Point E is the starting point of the receiver, point F is the ending point of the receiver’s trajectory, the red line is the receiver’s travel trajectory, and letter N and the white arrow are the north direction.

Experiment 2 may not fully represent densely built areas or areas obstructed by tall buildings. Therefore, to better verify the advantages of the proposed method in different urban environments. We have expanded the field tests to densely built areas or areas with tall obstacles, and added a real signal obstruction experiment 3. The scene of Experiment 3 is shown in Figure 17, where the receiver moves from point E to point F and the red line is the receiver’s travel trajectory. The data collection lasted for 207 s. It can be seen from the figure that there are many tall buildings distributed around the receiver’s trajectory. Therefore, the scene of Experiment 3 is suitable for dense buildings or tall occluded areas. During the Experiment 3 testing period, the total number of collected GNSS and cellular signals was about 3–5, with over 82% of the time being less than 4, which is consistent with the low observability scenario studied in this paper.

### 6.3. Experimental Results for Experiment 1

#### 6.3.1. Positioning Performance with Different Numbers of Signals

In this section, the extraction interval of measurements is set to 2 s, and a total of 150 epoch differential pseudorange measurements are used to verify the positioning performance of different numbers of signals and algorithms.

(1) $N^{bs}=2$ and $N^{gnss}=2$

At the beginning of the test, the receiver was stationary for 3 min and the change rate of the TOAs of the cellular SOPs was summarized. The clock drift estimation of the receiver was 0.0844 m/s. The altitude of the receiver was set to the height of BS 2, which is 77 m. These parameters were substituted into the CMWLS method, and the positioning results of the TWLS and Pei methods were compared with the proposed method.

**Figure 18:**
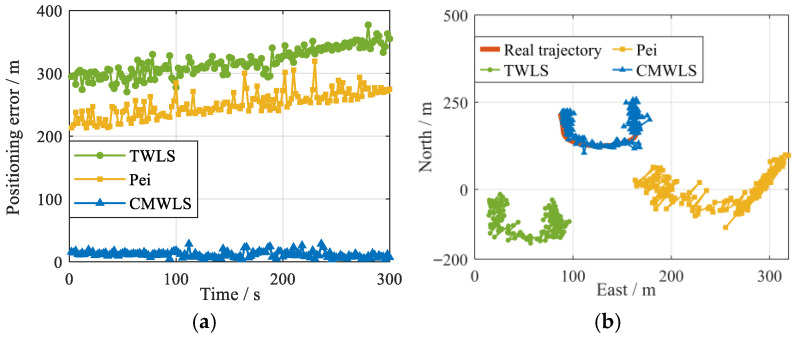
Performances of different algorithms under 2 GSs-2 BSs. (**a**) 3D Position RMSE; (**b**) 2D positioning trajectory.

The number of iterations was set to 10. For SVN (27, 16)-BS (2, 3), the positioning errors and trajectories of the three methods are shown in Figure 18. The positioning RMSEs and average errors are summarized in Table 4. From the results in the figure and table, it can be seen that the positioning performance of the proposed algorithm is significantly better than the other two methods with a positioning accuracy of up to 9.7 m.

(2) $N^{bs}=1$ and $N^{gnss}=2$

The TWLS and Pei methods cannot be used under the 2 GSs-1 BS scenario. In the CMWLS method, the positioning result of the CTWLS is used to initialize the PSDM model, and the minimum number of signals required is 3. In addition, in the CTWLS method, the constraint process is mainly reflected in the first step. Therefore, compared to the proposed CMWLS method, initializing the PSDM model using the positioning results of the first step of CTWLS may have poorer performance. For simplicity, this method is defined as CTWLS-1 initialization.

**Figure 19:**
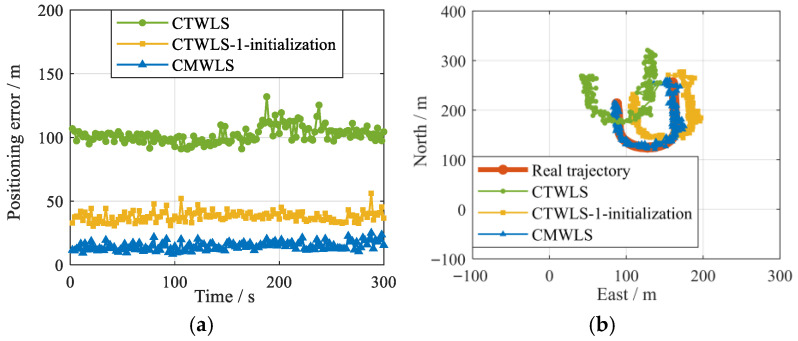
Performances of different algorithms under 2 GSs-1 BS. (**a**) 2D Position RMSE; (**b**) 2D positioning trajectory.

For the SVN (27, 16)-BS (1), the positioning errors and trajectories of the CMWLS, CTWLS, and CTWLS-1 initialization methods are shown in Figure 19. The RMSEs and average errors are shown in Table 5. It can be seen that when there are 1 BS and 2 GSs, the positioning performance of the proposed CMWLS method is superior to the other two methods. In addition, the positioning accuracy of PSDM initialized using CTWLS-1 is lower than that of the CTWLS initialization.

(3) $N^{bs}=2$ and $N^{gnss}=1$

**Figure 20:**
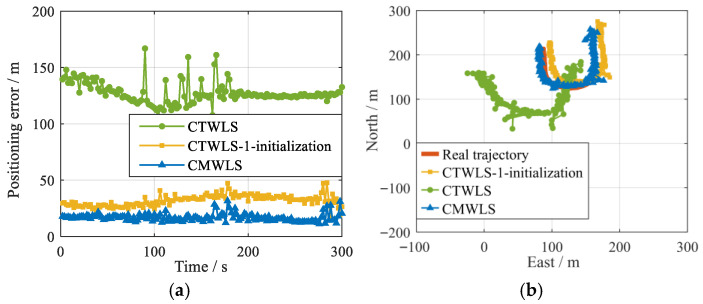
Performances of different algorithms under 1 GS-2 BSs. (**a**) 2D Position RMSE; (**b**) 2D positioning trajectory.

For the SVN (16)-BS (2, 3), the positioning errors and trajectories of the CMWLS, CTWLS, and CTWLS-1 initialization methods are shown in Figure 20. The RMSEs of the three positioning methods are shown in Table 6. According to the results, it can be seen that the performance of the proposed algorithm is superior to that of the other two methods with 1 GS-2 BSs. This is similar to the conclusion when there was 1 GSs and 2 BSs.

#### 6.3.2. Performance Analysis for Different Numbers of Epochs

**Figure 21:**
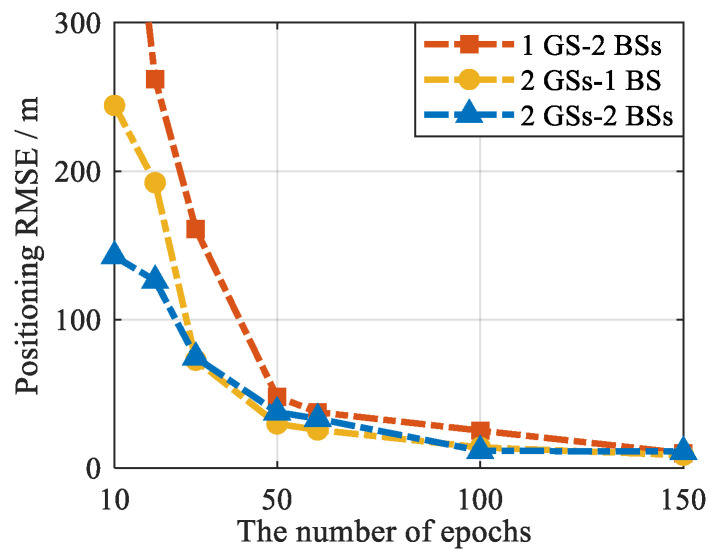
The positioning performance of the proposed algorithm under different epoch numbers.

The number of PSDM measurements is related to the number of signals and epochs. Variation in the number of epochs may affect the performance of the proposed algorithm. The performances of the proposed algorithm at different epoch numbers are shown in Figure 21. It can be seen from the results that as the number of epochs increases, the positioning accuracy of the proposed algorithm gradually improves.

#### 6.3.3. Performance of Initialization Constraints for Different Height Differences

The proposed method requires prior information about the receiver height when initializing constraints. Considering the small difference between the BS height and the receiver height, the proposed method initializes the constraint conditions using the BS height as an approximation of the receiver height. Therefore, the performance of the proposed method is related to the degree of approximation of the receiver altitude. To verify the effectiveness of this approximation, real signal experiments were conducted under different initialization constraints at different heights.

**Figure 22:**
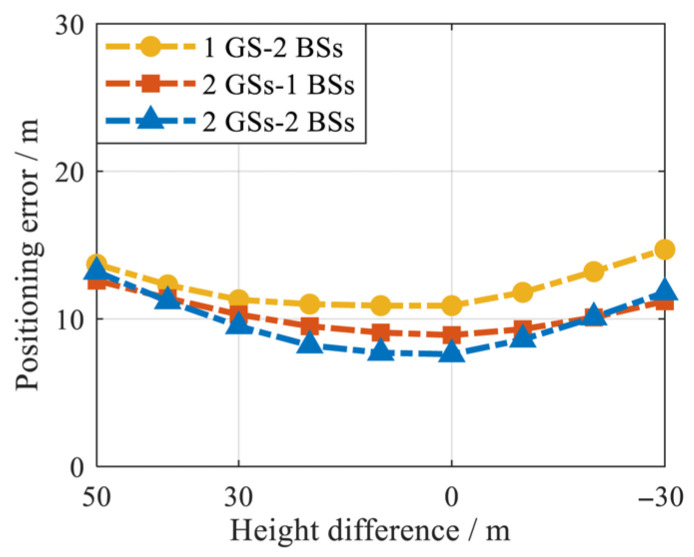
Positioning error of proposed methods under initialization constraints at different heights.

According to the actual location of the BSs in Table 3, the maximum height difference between a BS and a receiver is 48 m. Therefore, starting from a height difference of 50 m, the interval decreases by 10 m. The positioning RMSEs of the proposed method under different height initialization constraints are shown in Figure 22.

The results from Figure 22 show that the positioning accuracy of the proposed method improves as the height difference decreases. When the height difference is from 50 m to 0 m, the variation amplitude of the positioning error of the proposed method is basically within 5 m; i.e., the variability of height difference has a relatively small impact on positioning accuracy. Therefore, the proposed CMWLS algorithm is effective in initializing constraint conditions using the BS height as an approximation of the receiver height.

### 6.4. Experimental Results for Experiment 2

**Figure 23:**
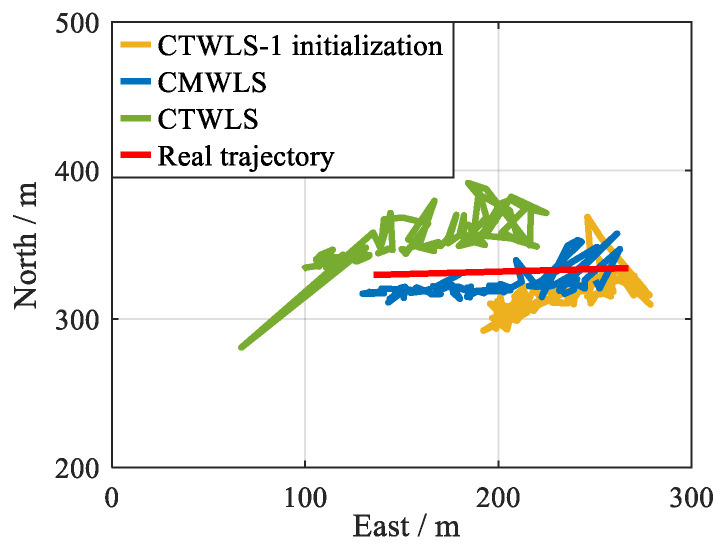
Positioning performance of different algorithms in real occlusion Experiment 2.

In the real occlusion experiment, since the number of visible signals may be less than four, the positioning performance of the proposed CMWLS method, CTWLS method, and CMWLS-1 initialization PSDM model is still compared. The positioning trajectories of the three methods are shown in Figure 23. Due to insufficient GNSS signals, accurate trajectory reference positions cannot be obtained through GNSS receivers. However, about 32 m south of the test section, the GNSS receiver can output positioning reference results. Due to the fact that the test route follows a straight path along the road, the position of the test trajectory can be roughly estimated based on this result as a reference. Although the reference position accuracy obtained through the above scheme is lower compared to that achieved through GNSS receivers, it can help to quantitatively analyze the positioning performance of different algorithms.

The positioning RMSEs and average error of the three methods are shown in Table 7. According to the results, it can be seen that the proposed PSDM fusion positioning model and CMWLS positioning method exhibit good positioning performance in real urban occlusion scenes, and do not require prior information on the initial position of the receiver.

### 6.5. Experimental Results for Experiment 3

**Figure 24:**
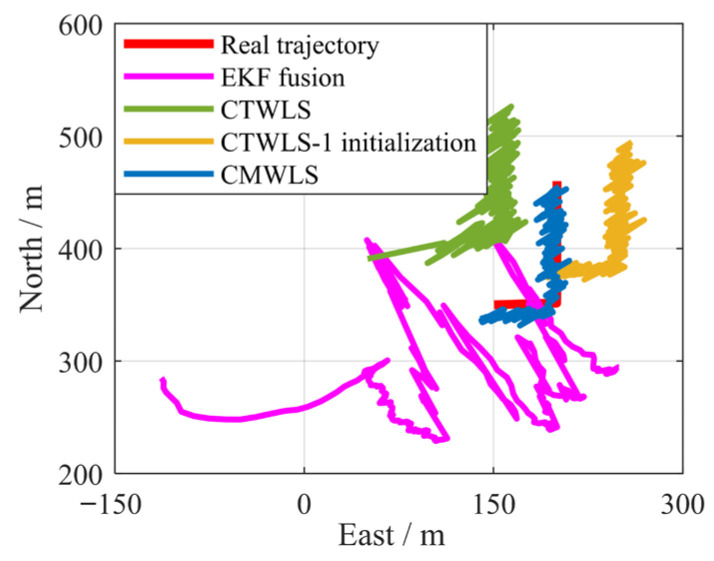
Positioning performance of different algorithms in real occlusion Experiment 3.

In the field of signal fusion, EKF is a common signal fusion positioning method. However, this method requires precise initial values for initialization. Otherwise, the positioning is prone to divergence. Therefore, we will compare the positioning performance of the proposed CMWLS method with EKF, CTWLS, and CTWLS-1 initialization methods. The EKF model is initialized by using the BS position as the initial receiver position. The positioning trajectories of four methods for Experiment 3 are shown in Figure 24. The positioning RMSEs of four methods are shown in Table 8. From the results in the figure and table, it can be seen that the proposed CMWLS method exhibits good positioning performance. Compared with the other three methods, the proposed method has lower positioning error. Among them, the EKF model is difficult to converge due to the lack of precise initialization. The above experimental results further validate the effectiveness and practicality of the proposed method in densely built urban areas.

## 7. Conclusions

In this paper, a PSDM positioning model was established to achieve cellular SOP–GNSS fusion. Three main issues have been resolved. Firstly, the asynchronous BS clock bias problem was solved through differential pseudorange measurements between different epochs. Secondly, a closed-form solution based on pseudo-linearization was derived for PSDM positioning, and the necessity for prior information of the receiver’s initial position in localization was removed. Thirdly, to reduce the minimum number of signals required for positioning and improve the global convergence of positioning, the pseudo-linearization matrix was reconstructed through variable separation, and the CMWLS method was proposed to solve the PSDM positioning model. Finally, the performance of the proposed fusion positioning method was verified and analyzed through simulation experiments and field tests. The experimental results of the field test show that (1) the positioning performance of the proposed method is superior to that of the TWLS, Pei, CTWLS, and CTWLS-1 initialization methods in low-observability environments, with a positioning accuracy of about 10 m, and (2) in real occlusion scenarios, the proposed method maintains better positioning performance compared to the other methods, and positioning accuracy was improved by approximately 64.5% and 57.7%. In summary, this research provides an effective solution method for maintaining the continuity of positioning services in low-observability environments without prior information regarding the initial receiver position.

In future work, we will focus on further enhancing the stability and adaptability of the proposed positioning method in more severe low-observability situations.

## Figures and Tables

**Table 1:** Simulation experiment configuration.

	BS Position/m	Satellite Position (on Field Test Data)
Number 1	(−100, −100, 101)	GPS SVN 16
Number 2	(500, 700, 77)	GPS SVN 27
Number 3	(100, 300, 81)	GPS SVN 4

**Table 2:** Positioning RMSEs for different positioning methods under different noise standard deviations.

	Noise: 0.01 m	Noise: 1 m	Noise: 5 m	Noise: 10 m
TWLS	0.37 m	6.2 m	193.7 m	376.4 m
Pei	0.31 m	5.3 m	121.6 m	235.9 m
CTWLS	0.21 m	3.7 m	9.4 m	136.2 m
CMWLS	0.17 m	0.89 m	6.7 m	18.6 m

**Table 3:** Information on LTE BSs.

No.	Position in ENU/m	Carrier Frequency	Bandwidth	CELL ID
1	(0, 0, 101)	2644.4 MHz	20 MHz	46
2	(5.4, 354.9, 77)	2017.5 MHz	15 MHz	7
3	(315.4, 343.9, 81)	2017.5 MHz	20 MHz	367
4	(82.2, −207.6, 73)	2330 MHz	20 MHz	260

**Table 4:** Positioning performance of the three methods under 2 GSs-2 BSs.

	CMWLS	TWLS	Pei
3D RMSE (m)	15.7	327.8	257.7
2D RMSE (m)	9.7	276.4	211.9
East RMSE (m)	5.9	73.5	118.7
North RMSE (m)	7.5	268.8	175.1
Up RMSE (m)	13.9	185.3	147.2

**Table 5:** Positioning performance of the three methods under 2 GSs-1 BS.

	CMWLS	CTWLS	CTWLS-1 Initialization
2D RMSE (m)	10.2	69.6	29.7
East RMSE (m)	6.9	34.4	22.9
North RMSE (m)	7.5	59.8	19.4

**Table 6:** Positioning performance of the three methods under 1 GS-2 BSs.

	CMWLS	CTWLS	CTWLS-1 Initialization
2D RMSE (m)	11.6	89.2	23.5
East RMSE (m)	7.5	62.6	15.2
North RMSE (m)	8.1	63.1	17.9

**Table 7:** Positioning errors of different algorithms in a real occlusion scene Experiment 2.

	CMWLS	CTWLS	CTWLS-1 Initialization
2D RMSE (m)	18.9	53.2	43.5
2D mean error (m)	18.2	51.6	41.8

**Table 8:** Positioning errors of different algorithms in a real occlusion Experiment 3.

	CMWLS	CTWLS-1 Initialization	CTWLS	EKF
2D RMSE (m)	16.5	51.3	59.7	179.2
2D mean error (m)	15.2	47.6	56.1	47.6

## Data Availability

Data is contain within the article.
